# Cortical asymmetries at different spatial hierarchies relate to phonological processing ability

**DOI:** 10.1371/journal.pbio.3001591

**Published:** 2022-04-05

**Authors:** Mark A. Eckert, Kenneth I. Vaden, Federico Iuricich, Dyslexia Data Consortium

**Affiliations:** 1 Hearing Research Program, Department of Otolaryngology—Head and Neck Surgery, Medical University of South Carolina, Charleston, South Carolina, United States of America; 2 Visual Computing Division, School of Computing, Clemson University, Clemson, South Carolina, United States of America; "Universite Libre de Bruxelles", BELGIUM

## Abstract

The ability to map speech sounds to corresponding letters is critical for establishing proficient reading. People vary in this phonological processing ability, which has been hypothesized to result from variation in hemispheric asymmetries within brain regions that support language. A cerebral lateralization hypothesis predicts that more asymmetric brain structures facilitate the development of foundational reading skills like phonological processing. That is, structural asymmetries are predicted to linearly increase with ability. In contrast, a canalization hypothesis predicts that asymmetries constrain behavioral performance within a normal range. That is, structural asymmetries are predicted to quadratically relate to phonological processing, with average phonological processing occurring in people with the most asymmetric structures. These predictions were examined in relatively large samples of children (*N =* 424) and adults (*N* = 300), using a topological asymmetry analysis of T1-weighted brain images and a decoding measure of phonological processing. There was limited evidence of structural asymmetry and phonological decoding associations in classic language-related brain regions. However, and in modest support of the cerebral lateralization hypothesis, small to medium effect sizes were observed where phonological decoding accuracy increased with the magnitude of the largest structural asymmetry across left hemisphere cortical regions, but not right hemisphere cortical regions, for both the adult and pediatric samples. In support of the canalization hypothesis, small to medium effect sizes were observed where phonological decoding in the normal range was associated with increased asymmetries in specific cortical regions for both the adult and pediatric samples, which included performance monitoring and motor planning brain regions that contribute to oral and written language functions. Thus, the relevance of each hypothesis to phonological decoding may depend on the scale of brain organization.

## Introduction

The ability to map letters to corresponding speech sounds, or phonological processing, is crucial for becoming a proficient reader [1–3]. There is substantial variation in phonological processing abilities due, in part, to genetic influences [4,5]. Individual differences in phonological processing have also been related to varied brain structure [6–10], including within a well-established set of cortical regions that support phonological processing [11–13]. The degree to which these regions exhibit hemispheric asymmetries in structure and/or function has been proposed to explain individual differences in language abilities, including phonological processing.

The human brain exhibits pronounced asymmetries [14–16], including within brain regions that influence phonological processing accuracy [17,18]. These regions exhibit more left than right hemisphere activity during phonological processing [19,20]. Specifically, phonological processing tasks (e.g., rhyme decision) elicit increased leftward activity in supramarginal, inferior frontal, superior temporal, and dorsal cingulate gyri (as shown in https://www.neurosynth.org/analyses/terms/phonological [21]). Although it is not clear that the extent of functional asymmetries depends on the extent of structural asymmetries [22–24], these brain regions are larger in the left compared to the right hemisphere [15,25]. That is, they exhibit leftward structural asymmetries.

Leftward asymmetry in the size of the superior temporal gyrus, in particular, is classically thought to reflect leftward hemispheric organization for language [26–28], which, when disrupted, contributes to poor reading skills according to a cerebral lateralization hypothesis [29–31]. This idea is consistent with a larger animal literature indicating that lateralization provides performance benefit through increased perceptual discrimination and processing capacity [32]. There is some evidence that atypical leftward asymmetries influence reading skills [33–35], including findings that more leftward structural asymmetries in language-related brain regions relate to better oral language abilities [36–39]. However, associations between atypical structural asymmetries and reading disability are often small in effect size ([34,40]; for review [33]) and inconsistently replicated ([34,39–43]; for review [44]), which may be due to demographics [34,37], type of language disability [45–47], differences in planum measurement criteria [33,48], or approaches that are focused on single structures rather than the contribution of asymmetries across cortical regions that collectively support phonological processing [11–13].

Lateralization does not always occur with optimal performance or outcomes [32]. For example, participants with typical leftward asymmetry of the arcuate fasciculus had verbal memory performance in the average range compared to those with arcuate fasciculus symmetry and above average verbal memory [49]. Or, for example, participants with more leftward or rightward laterality during visual-half field testing of 1-back word recognition had lower performance than those with less laterality [50]. These and other findings [51] suggest that associations between cerebral asymmetries and behavior can be task dependent, and/or that asymmetries provide for effective performance at the population level, but that the magnitude of asymmetry does not increase linearly with performance. That is, asymmetries may provide a constraint to ensure effective phonological processing at the population level or canalize the range of behavior within the normal range [52].

Canalization, visualized in metaphor by Conrad Waddington as a ball rolling along a grooved slope to maintain a predetermined developmental trajectory [53], is hypothesized to guide or buffer the normal expression of a phenotype from disruptions in development [54,55] through protective genetic mechanisms [56,57] and/or experience [58]. Whereas more exaggerated structural asymmetries may preserve the expression of normal phonological processing (that is, in the presence of dyslexia risk alleles), the absence of asymmetries would allow for the unconstrained expression of impaired-to-exceptional abilities. This buffering idea was presented as an explanation for above average reading comprehension in university students with a rightward shift away from the modal distribution of leftward planum temporale asymmetry [52]. However, there appears to be relatively limited consideration of canalization in neuroimaging studies of language and related cognitive functions.

### Experimental goals and hypothesis testing

**Fig 1:**
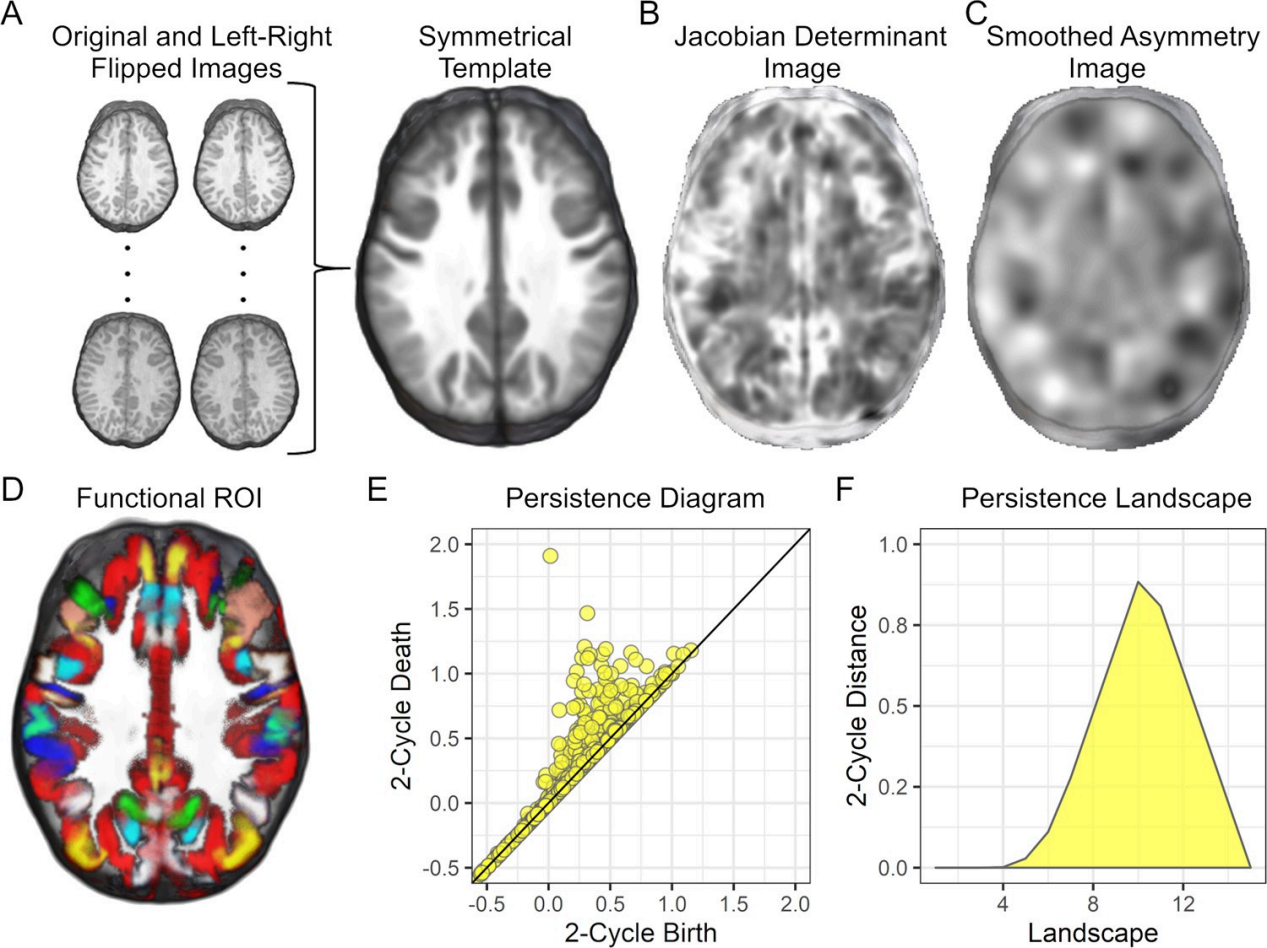
Imaging methods. **(A)** T1-weighted images and a left–right flipped copy of each image were used to create a symmetrical study-specific template. **(B)** Each native space T1-weighted image was normalized to the symmetrical template to create Jacobian determinant images representing volumetric expansion and contraction of brain regions to fit to the template [45]. **(C)** Each log Jacobian image was smoothed, and homologous left and right hemisphere voxel values were subtracted to create asymmetry images. **(D)** The Human Connectome multimodal parcellation (180 ROI) were used to define the analysis space as functional imaging data were not available for this multisite sample. Each color represents a different ROI. (**E)** For each ROI, we computed a persistence diagram limited to 3D asymmetry structures called 2-cycles or hyperintense structures rather than hypointense voids in the image. A persistence diagram is shown for 2-cycles collected from within all left-hemisphere ROI for the participant’s image data shown in **(C)**. Each yellow circle represents a distinct asymmetric structure. The x-axis birth refers to the lower contrast boundary of the asymmetric structure and the y-axis death refers to peak contrast or degree of asymmetry. **(F)** The persistence diagram was transformed to a persistence landscape that represented the maximal asymmetry or contrast for each lower contrast value that defined the boundary of the asymmetry. The data from each landscape position were then used to examine the cerebral lateralization and canalization hypotheses. Underlying data and code for plots E and F: https://osf.io/75g9d. ROI, region of interest.

To test the hypotheses that structural asymmetries (1) confer a phonological processing advantage or (2) canalize (produce average performance), the directional magnitude of left-to-right structural asymmetries within each cerebral hemisphere was measured using T1-weighted images of the brain from pediatric (*n =* 424) and adult (*n* = 300) samples (Table A in S1 Appendix). Asymmetry images were created using an established deformation-based method that defines asymmetries as the amount of volumetric expansion and contraction that was necessary to align voxels in each brain image to a symmetrical brain template (Fig 1A–1C) [59]. For example, a leftward asymmetry would be observed when the left motor cortex is reduced in volume to fit to the symmetrical template and the right motor cortex is increased in volume to fit to the symmetrical template. Cortical regions that are leftward or rightward asymmetric in these images exhibit hyperintense voxel values (e.g., motor/somatosensory cortex, planum temporale, and frontal and occipital petalias), which can be quantified using persistent homology within functionally distinct cortical parcellations or regions of interest (ROIs; Fig 1D) [60,61].

Persistent homology is a mathematical approach for defining topological features or, in this study, 3D asymmetries that appear as bright objects in the images. Each asymmetric object within an ROI is represented in a persistence diagram by its lower intensity boundary value (birth) and higher intensity peak value (death) (Fig 1E), or how long a structure persisted across a range of asymmetry values and thus its magnitude of asymmetry. This approach for identifying asymmetric structures is like varying the exposure time when taking a photograph. Different objects in a scene will appear (birth) and then blend with the background (death) across exposure times. Although the number of asymmetric structures within ROI can vary, the data can be analyzed across participants by transforming the persistence diagram into a persistence landscape that represents the most asymmetric object across the range of lower intensity boundary (birth) values (Fig 1F) [61]. Thus, the persistent homology data can be considered a form of data reduction, as shown in Fig A in S1 Appendix where the persistent homology measure of dorsal cingulate/paracingulate asymmetry was significantly related to voxel-level data in the same region.

Persistence landscape data were used to examine whether individual differences in structural asymmetries across each hemisphere and within each ROI were significantly related to phonological processing and, specifically, a phonological decoding measure of the ability to sound out a series of orthographically presented pseudowords as accurately as possible. Phonological processing was examined because this construct, represented here by a phonological decoding measure, is a strong predictor of reading proficiency [1], and impairments can persist into adulthood [62–64]. Moreover, preliterate phonological processing ability has been related to individual differences in left hemisphere structure ([65–67]; although not asymmetry in [67]), has high heritability [5], and has been linked to genes that are expressed in classic language-related regions of the left cerebral hemisphere [68]. That is, we examined phonological processing in this study because it is a foundational ability for proficient reading that may be influenced by structural asymmetries.

Tests of the cerebral asymmetry and canalization hypotheses focused on identifying effects that persisted across development into early adulthood and that are replicable. Based on the cerebral asymmetry hypothesis, above average phonological processing was predicted to occur with a greater extent of leftward structural asymmetry, particularly in brain regions that support phonological processing. Based on the canalization hypothesis, average phonological decoding was predicted to occur with a greater extent of leftward or rightward asymmetries. Results were considered significant (*p* < 0.05) when present in pediatric (*N =* 424; mean age = 10.54 years) and adult (*N* = 300; age: mean age 20.86 years) datasets. Significant results were observed for each hypothesis but at different spatial scales of brain organization.

## Results

### Test of the cerebral lateralization hypothesis

**Fig 2:**
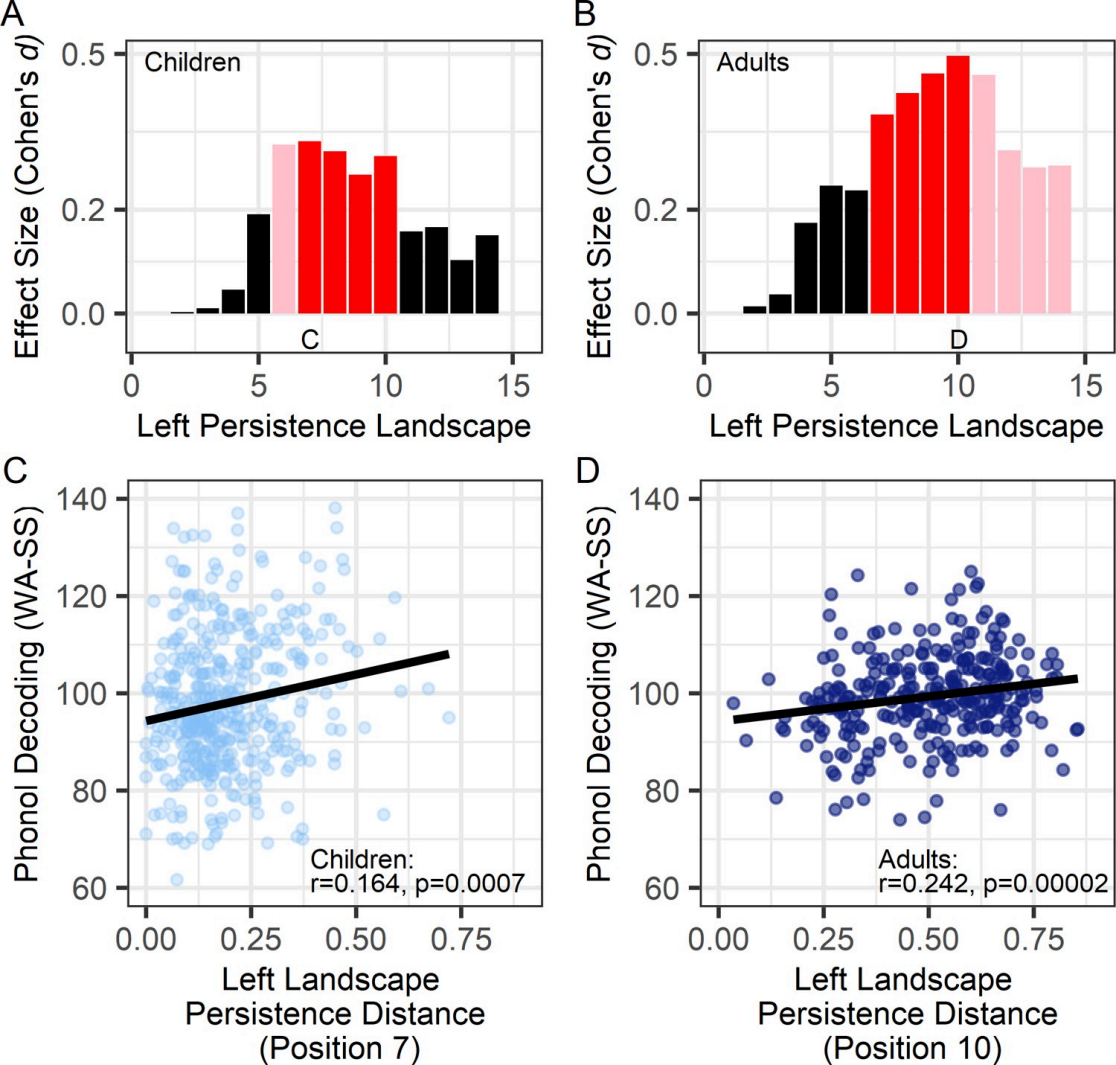
Group results show that structural asymmetries across the left hemisphere were significantly related to phonological decoding accuracy. **(A, B)** Bar plots show that after covarying for effects of age, sex, and research site, structural asymmetries across left hemisphere ROI (maximal asymmetry) exhibited small to medium effect size associations with phonological decoding in (**A**) children and (**B**) adults. These effects were considered significant when present in both samples at the same landscape position (red: *p* < 0.05 in both samples; pink: *p* < 0.05 in just that sample). The C and D labels indicate the largest effects that are plotted in (**C, D**) where the associations between structural asymmetries and decoding are shown (note that phonological decoding residual values are presented after accounting for age, sex, and site effects based on linear regression). Phonol Decoding (WA-SS)—Word Attack Phonological Decoding standard scores. Persistence distance refers to the death–birth life span of the asymmetric structures. Underlying data and code for plots A-D: https://osf.io/75g9d. ROI, region of interest.

Within each cortical ROI, including language-related regions, there were nonsignificant associations between leftward or rightward asymmetry and phonological decoding (Figs B and C in S1 Appendix). However, children and adults with the most exaggerated asymmetry across the left hemisphere ROI had higher phonological decoding accuracy (Fig 2A and 2B), based on linear regression analyses that included 10,000 bootstrap samples [e.g., Fig 2C, pediatric: *t*_(420)_ = 3.40 (CI_95%_ = 1.29 to 5.52), *p* = 0.0007, Cohen’s *d* = 0.332; Fig 2D, adults: *t*_(297)_ = 3.91 (CI_95%_ = 0.95 to 5.32), *p* = 0.0001, Cohen’s *d* = 0.382]. That is, at multiple landscape positions, there were significant associations between phonological decoding and the magnitude of asymmetry for the children and adults. These effects were present when total gray matter and white matter volume (total brain volume) were included in the regression models (Table B in S1 Appendix). The nonsignificant right hemisphere effects were small and inconsistent across pediatric and adult samples (Figs DA and DB in S1 Appendix).

The associations between phonological decoding and left hemisphere asymmetry appeared to be due asymmetries that were bounded by regions of hemispheric symmetry and had relatively high peak asymmetry values (e.g., the point in Fig 1E furthest from the diagonal). The landscape and phonological decoding associations shown in Fig 2C and 2D remained significant when limiting the analyses within the range of these large asymmetries (persistence diagram: birth > 0.00 to < 0.20 and death > 1; children: *r* = 0.114, *p* = 0.019; adults: *r* = 0.23, *p* = 5.72 e-05). In contrast, these effects were diminished when restricting the analyses to leftward asymmetries with higher boundary values (birth > 0.20; children: *r* = 0.07, *p* = 0.136; adults: *r* = −0.01, *p* = 0.775). Thus, the phonological decoding associations were due to structures with a broad range of asymmetry values rather than structures with extreme asymmetries values within a larger asymmetry.

### Cerebral lateralization effects: Behavioral specificity

We next considered the behavioral specificity of the phonological decoding and structural asymmetry association shown in Fig 2C and 2D. The significance of these effects was largely unaffected after controlling for variance in phonological decoding related to Verbal IQ, reading comprehension, or rapid naming (of letter or number strings) (Cohen’s *d* > 0.23, *p* ≤ 0.02 across both samples). Controlling for real word identification accuracy did affect the significance of phonological decoding associations with left hemisphere asymmetry (pediatric: Cohen’s *d* = 0.16, *p* = 0.107; adults: Cohen’s *d* = 0.40, *p* = 0.0006), because the phonological decoding and real word identification measures were so strongly related (pediatric: *r* = 0.86, *p* = 4.0E-126; adults: *r* = 0.60, *p* = 2.86E-30). Table C in S1 Appendix presents the association between leftward asymmetry and each behavioral variable, and Table D in S1 Appendix presents the Pearson correlations between each behavioral variable.

### Cerebral lateralization effects: Methodological specificity

Group-level independent component analysis (ICA) can also be considered a data reduction approach and was used here to determine the extent to which common patterns of asymmetry across the cerebrum were associated with phonological decoding. Two spatial patterns of covarying asymmetries were observed in the pediatric and adult samples (Fig E in S1 Appendix), which included variation in a left occipital region and medial Heschl’s gyrus/planum temporale region that exhibit a leftward asymmetry across participants in voxel-based analyses (Fig F in S1 Appendix). However, the independent component weights representing the degree of asymmetry in each region did not exhibit significant linear associations with phonological decoding (Cohen’s *d*s < 0.15, *p*s > 0.12 across both samples). These ICA results further indicate that the persistence landscape results were not dependent on structural asymmetries within those brain regions that typically exhibit the most pronounced asymmetries [that is, anterior insula, medial planum temporale, or occipital petalia (Figs E and F in S1 Appendix)].

In summary, these results do not support the hypothesis that leftward asymmetry of a specific brain region predicts phonological processing accuracy. However, the most leftward asymmetry across all of the left hemisphere ROI, which allowed for the brain region exhibiting the most asymmetry to vary across participants, was significantly related to better phonological decoding. This latter set of exploratory results, which replicated across samples, was most pronounced for individual differences in the ability to map speech sounds to letters compared to other reading related abilities.

### Test of the canalization hypothesis

**Fig 3:**
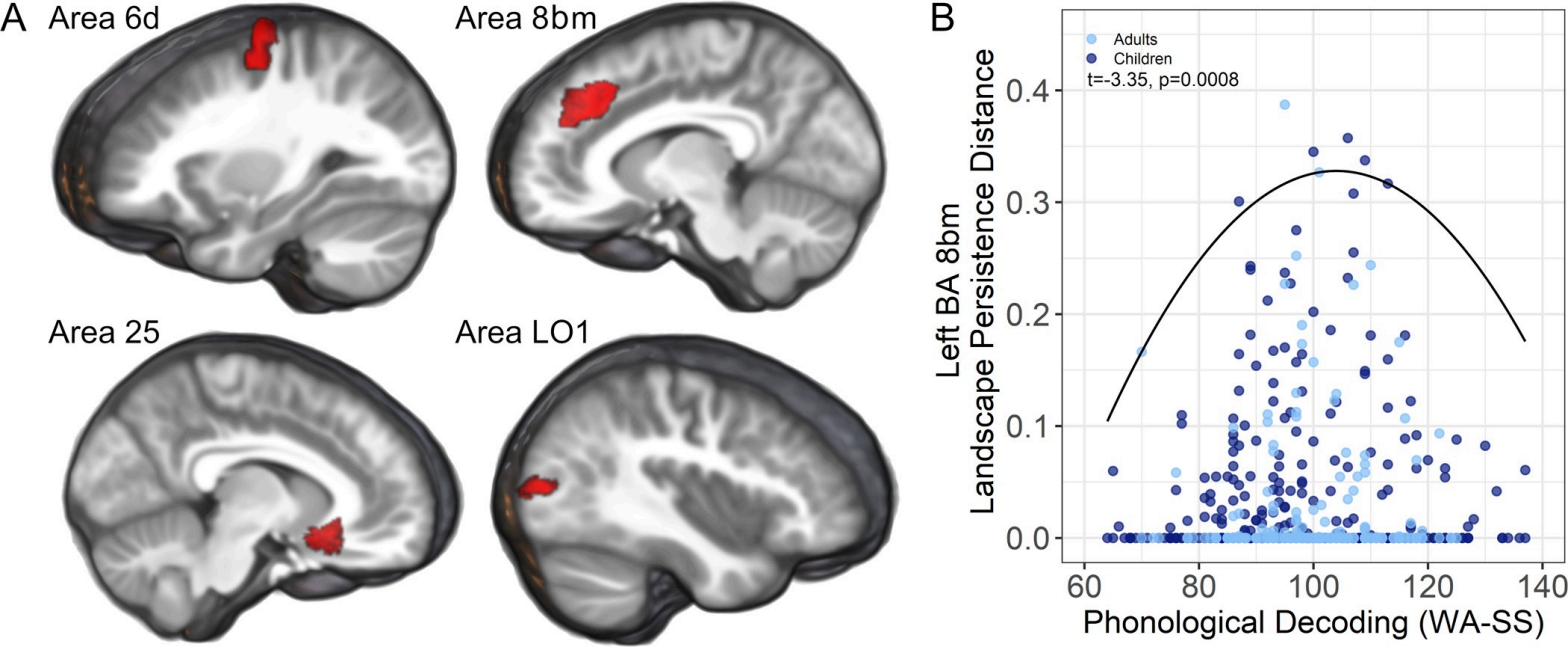
Cortical regions where structural asymmetries appeared to canalize phonological decoding. **(A)** Children and adults who had more structural asymmetries in each region were more likely to have phonological decoding in the average range, whereas those with low to high range of performance had no or minimal asymmetries in these regions. Top row: left hemisphere; Bottom row: right hemisphere. **(B)** An example of this 99th percentile quantile effect is plotted for BA 8bm, where the fitted effect is shown for an analysis across both samples. To further verify that outliers could not influence these results, 4 children with the most pronounced asymmetries (>.5 on the y-axis) were excluded from the combined group results shown here. Images and ROI shown in A and the underlying data and code for plot B: https://osf.io/75g9d. BA, Brodmann area; ROI, region of interest.

We then tested the hypothesis that structural asymmetries canalize the expression of reading skills. Again, more asymmetric structures were predicted to occur in participants with phonological decoding that was in the average range, while those with less asymmetry were expected to demonstrate below to above average phonological decoding. This prediction was tested using a quadratic quantile regression (tau = 0.99), where negative estimates represent the predicted inverted-U sandpile association between phonological decoding and asymmetries. There were no significant associations with phonological decoding accuracy when examining asymmetries across left or right hemisphere gray matter regions (Fig G in S1 Appendix). However, phonological decoding accuracy exhibited significant associations within specific brain regions for the pediatric and adult samples, as shown in Fig 3A. Inverted-U quantile regression results are shown for all ROI in Fig H in S1 Appendix. Here, more exaggerated asymmetry occurred with average phonological decoding accuracy.

### Canalization effects: Behavioral specificity

Evidence for canalization included the left hemisphere Brodmann area 8bm or dorsal cingulate/paracingulate. Children and adults with more leftward asymmetry in the dorsal cingulate/paracingulate had phonological decoding that was in the average range, as shown in Fig 3B. These effects were not dependent on total brain volume (Table E in S1 Appendix). However, and in contrast to the cerebral lateralization hypothesis analyses, they were no longer significant after accounting for variance in phonological decoding related to Verbal IQ, reading comprehension, rapid naming, or real word identification (maximum Cohen’s *d* = 0.13, *p* = 0.171). Fig I in S1 Appendix shows the dorsal cingulate/paracingulate association between real word decoding/reading, passage comprehension, verbal comprehension, and rapid naming.

### Cerebral lateralization effects: Methodological specificity

Canalization is a long-standing developmental hypothesis with limited supporting evidence in the neuroimaging literature. The persistent homology measure of structural asymmetries may have been particularly sensitive to canalization effects because it summarized the maximum asymmetry originating within a region rather than the average degree of asymmetry across the ROI, which is a measure that can be influenced by asymmetries originating outside of an ROI. We examined the extent to which evidence for canalization could also be seen when averaging voxel values within the ROI. The average asymmetry across all voxels within the dorsal cingulate/paracingulate was not significantly associated with phonological decoding [Children: *t* = 1.11 (CI_95%_ = −0.78 to 2.98), *p* = 0.268, Cohen’s *d* = 0.11; Adults: *t* = −0.36 (CI_95%_ = −2.83 to1.73), *p* = 0.718, Cohen’s *d* = −0.04]. Thus, the maximum asymmetry of structures originating in an ROI, rather than the average magnitude of asymmetry across a larger anatomical space, influenced the canalization results.

## Discussion

Results from this study show that individual differences in phonological decoding relate to structural brain asymmetries in two distinct patterns. First, leftward asymmetry linearly predicted higher phonological decoding accuracy, but only when considering the most asymmetric structure across the left hemisphere rather than within specific brain regions. Thus, the most asymmetric structure across the left cerebral hemisphere, but not the right hemisphere and not specific language-related brain regions, appeared to facilitate phonological decoding. Second, relatively pronounced structural asymmetries within specific brain regions, which included regions that support domain general functions, were observed for participants with phonological decoding in the normal range in comparison to those with above or below average phonological decoding. That is, structural asymmetries in specific brain regions appeared to constrain phonological decoding performance within the normal range. Together, this pattern of results is consistent with cerebral lateralization and canalization hypotheses, which appear to have relevance at different scales of brain organization.

### Mixed support for the cerebral lateralization hypothesis

Hemispheric asymmetries are widely thought to confer a processing advantage through specialization of functions, particularly when an animal must process different types of information simultaneously [69]. The cerebral lateralization hypothesis proposes that left hemisphere representation for language, due to structural asymmetries, confers a language processing advantage. There was not strong evidence that spatially specific cortical regions exhibited increased leftward asymmetry or rightward asymmetry with increased phonological decoding. Instead, the maximum asymmetry across the left hemisphere was related to phonological decoding without apparent spatial specificity. This result is thus only partially consistent with the prediction that language-related cortical regions [19,20] would exhibit greater leftward asymmetries in children and adults with above average phonological processing accuracy.

The planum temporale is often a focus in the study of structural and functional asymmetries, including studies of impaired phonological processing. For example, atypical planum temporale symmetry was reported in children who had poor reading skills and a family history of reading disability [35]. While we did not observe significant effects in the planum temporale, quantile regression (tau = 0.5) demonstrated that children with poorer phonological decoding exhibited more rightward asymmetry in the right parabelt (PBelt) ROI (Fig B in S1 Appendix). A post hoc analysis demonstrated that this effect was present in male but not in female children, which is consistent with evidence that planum temporale asymmetry relates to dyslexia in males rather than in females [34]. The same results were not observed in adults, however, which may be consistent with small effects in the literature for surface area measures of the planum temporale [33]. It is possible that limited family history and handedness information could explain inconsistent results between samples in the current study or that relations between planum morphology and the phonological decoding measure used in the current study are more likely in children than in adults. As discussed later, we also note that the deformation-based asymmetry approach used in the current study differs from manual measures of planum temporale surface area in important ways, including the influence of sulcal/gyral variability that influence measures of the planum temporale.

The most consistent support for the cerebral lateralization hypothesis was observed when considering asymmetries across the left hemisphere. The persistent homology approach appeared to be sensitive to asymmetry effects that were not constrained to a spatially specific group of voxels or functionally defined region of interest. Moreover, the effects in this study appeared to depend on asymmetries that were bounded by symmetrical regions, based on limiting the analyses to relatively low persistence birth values. That is, effects appeared to be due to discrete structural asymmetries and were not due to random variation in the extent of asymmetry within an asymmetric structure.

The current findings may be consistent with a network perspective rather than a modular view that asymmetry in a specific language-related structure underlies speech sound processing abilities. A resting state fMRI study demonstrated that left hemisphere cortical regions exhibited greater connectivity (stronger time series correlations) compared to right hemisphere cortical regions, and a greater degree of this within (left) hemisphere connectivity related to greater vocabulary knowledge [70]. This result is consistent with evidence for a left hemisphere network that provides feedback to auditory cortex during phonological processing [71]. We hypothesize that the degree of left hemisphere functional network asymmetry and association with performance is influenced by the most leftward asymmetric cortical region. Here, the most asymmetric region would be a constraint that determines the exclusivity of left hemisphere processing during phonological processing. That is, leftward structural asymmetry may influence phonological processing through the coordination of activity across the left hemisphere. To our knowledge, this testable hypothesis has not been examined. Given the correlational nature of our results, however, associations between spatially nonspecific asymmetry and phonological decoding may instead reflect the experiential consequences of leveraging different brain regions when learning to read [72,73].

### Support for the canalization hypothesis

Canalization is hypothesized to ensure the normal expression of a phenotype and a normal distribution or population-level consistency in behavior. There is limited evidence for brain structure canalizing behavior, however. Conventional neuroimaging studies involving linear voxel-based analyses would of course not be expected to have sensitivity to nonlinear canalization effects across multiple voxels. This study was specifically designed to test for canalization effects using a nonlinear statistical analysis that evaluated the degree to which participants with the most pronounced asymmetries were more likely to have performance in the normal range. Unexpectedly, canalization effects were observed within specific brain regions that may provide support but are not considered to be essential for processing phonologic and orthographic information [17,20].

The canalization results occurred in regions that exhibit elevated activity during oral and written language tasks [74], including a dorsal cingulate/paracingulate region that has been shown in many studies to have a role in cognitive control, value-based decision-making, and motor planning [75,76] during the performance of verbal and nonverbal tasks [77]. For example, dorsal cingulate activity has been associated with tracking task conditions to evaluate and guide performance [78], including for speech–sound discrimination and rhyme generation tasks [79,80]. This performance monitoring function appears to contribute to adjustments in decision-making [81,82] with the goal of optimizing task performance, as suggested by elevated prestimulus activity in the dorsal cingulate/paracingulate prior to accurate word recognition, for example [83,84]. Thus, domain general functions of dorsal cingulate/paracingulate and perhaps BA 6d premotor cortex may serve to canalize or maintain the normal expression of behavior through goal-directed top-down control.

Domain-general executive function(s) could support the normal expression of phonological decoding accuracy through more cautious decision-making, thereby ensuring that sufficient information is accumulated to make a fully informed response [85]. Indeed, increased response caution has been observed in children with reading disability [86], and increased activity was observed in the left anterior cingulate among young adults who appeared to have compensated for their reading disability compared to people with persistent reading disability [87]. However, people with leftward paracingulate sulcus asymmetry can also exhibit shorter reaction times during response inhibition tasks [88], which may limit optimal performance in challenging task conditions when response caution is needed [89]. That is, faster responding may be advantageous for relatively easier test items but also limit performance when response inhibition and caution may be necessary for harder items (that is, for longer pseudowords) and thus result in performance that is in the average range. Above-average performance may occur in people with symmetrical dorsal cingulate cortex because of increased response inhibition from the right lateral frontal cortex [90]. This prediction is consistent with evidence that children with better phonological processing skills have higher response inhibition accuracy [91,92]. Clearly, additional study is necessary, including with phonological processing tasks with time constraints, to determine how domain general brain regions that support task performance may canalize behavior.

### Experimental design and limitations

The use of 2 relatively large samples provided an opportunity to detect small but consistent structural asymmetry effects. Statistically significant results in the current study were defined as effects (*p* < 0.05) that were present in the pediatric and adult samples, thus ensuring that each effect was replicable. In addition, we sought to control for differences that could arise from sampling and image acquisition across research sites, sex/gender groups, and age given the broad age range of the samples. However, the small the medium effect size correlations observed in this study precludes inference about direct causation.

This study was also designed to examine structural asymmetries that are present early in development, including the Sylvian fissure [93] and surrounding cortical regions [94]. We note that there did not appear to be strong effects of age on the standardized phonological decoding measure (linear *r*_(422)_ = −0.07, *p* = 0.16; quadratic *r*_(422)_ = −0.05, *p* = 0.32; cubic *r*_(422)_ = −0.03, *p* = 0.55) nor the hemispheric asymmetry measures (e.g., left hemisphere asymmetries: linear *r*_(422)_ = 0.02, *p* = 0.71; quadratic *r*_(422)_ = 0.02, *p* = 0.75; cubic *r*_(422)_ = −0.01, *p* = 0.83). Moreover, we statistically controlled for age in the regression analyses to ensure that effects would be replicable across pediatric and adult samples. This approach would have limited sensitivity to effects in specific brain regions, perhaps including the superior temporal sulcus where we previously observed a rightward topological asymmetry that increased with age in a pediatric sample [61]. It is possible that there are developmental periods where asymmetries in specific cortical regions are predictive of phonological processing (or vice versa) because of age and experience-related changes in brain asymmetries or behavior, although structural asymmetries and socioeconomic status can exhibit independent additive effects in predicting phonological processing measures in children [33].

We also considered the relative behavioral specificity of the phonological decoding results. Although statistical control for differences in real word reading resulted in a nonsignificant association between phonological decoding and left hemisphere structural asymmetry relationship in the children, the phonological decoding association with the left hemisphere structural asymmetry was largely unaffected after controlling for other reading and language abilities. This observation may be consistent with evidence that phonological processing is a foundational skill for the development of other reading and language abilities [1–3,95] and enduring for people with reading disability [62–64], but less influential on the acquisition of lexical knowledge in proficient readers, as demonstrated by the observation that phonological decoding was relatively more strongly associated with real word reading in the children compared to the adults. In contrast, the phonological decoding association with structural asymmetry of the dorsal cingulate/paracingulate (Brodmann area 8bm) was nonsignificant after controlling for the other reading and language measures, which may be consistent with the contribution of these domain general regions to a wide range of cognitive functions. This canalization result may also indicate that large sample sizes are needed for analysis focused on asymmetry data at the 99th percentile across the range of phonological decoding scores. Finally, the Word Attack measure of phonological decoding was the most consistently shared measure of the ability to manipulate speech sounds in this multisite retrospective study. It is unclear if other phonological processing measures, such as a phonological working memory or removing and blending speech sounds, would exhibit similar effects.

### Image analysis approaches

The methods used in this study, including the relatively novel persistent homology approach, provided new insights into the association between structural asymmetries and behavior in comparison to conventional voxel-based and volume averaging approaches. The volume averaging approach is often used with the goal of reducing noise at the voxel level, in addition to reducing the number of statistical comparisons. This approach can be problematic if sources of anatomical variance outside of an ROI affect the values within the ROI or if there are unique patterns of variance within an ROI that are averaged together. Nonsignificant phonological decoding associations were observed when averaging all voxel values within left dorsal cingulate/paracingulate, premotor, and lateral occipital ROI. This difference in results between voxel-wise averaging and persistent homology approaches could be due to the influence of asymmetries originating outside of an ROI (e.g., a spatially large asymmetry with a maximum value in motor cortex, shown in Figs E and F in S1 Appendix, which extends into the Brodmann area 6d ROI shown in Fig 3A). This would affect the averaged voxel-wise measure (e.g., of Brodmann area 6d) but not the persistent homology measure because the maximum voxel value of an asymmetry had to occur within an ROI to be measured with the persistent homology approach.

There were also nonsignificant associations between the ICA estimates of volumetric asymmetries and phonological decoding, which suggested that the persistent homology associations with phonological decoding were not driven by brain regions that exhibit the most pronounced and consistent asymmetries (e.g., leftward occipital petalia). Instead, the persistent homology results appeared to depend on qualitatively distinct asymmetries that appeared to be due to hemispheric differences in sulcal/gyral variability, as in the case of the dorsal cingulate results, and perhaps increased cortical surface area given the consistency in asymmetry results from surface area and deformation-based approaches (e.g., results shown in [25] and Fig F in S1 Appendix). That is, the persistent homology approach appeared to characterize hemispheric differences in sulcal/gyral features (Fig J in S1 Appendix) and volume [61] across the persistence diagram. It may have been feasible to select participants with the same sulcal/gyral features to examine spatially specific volumetric effects with the deformation-based approach, but this would likely have missed effects from the current study that appeared to be influenced by differences in sulcal patterning. Availability of the code used in this study should facilitate comparison of imaging methods, the examination of how asymmetries and specific language function may depend on demographic variables, and replication of our findings.

## Conclusions

Linear and nonlinear associations between structural asymmetries and phonological decoding accuracy were observed with small to medium effect sizes in this study of 424 children and 300 adults. To the extent that structural asymmetries have a causal role in the development of phonological processing skills rather than reflecting the consequence of atypical language development [96], the results of this study suggest the need to reframe explanations for how structural asymmetries underlie the expression of oral and written language abilities. Cerebral lateralization and canalization hypotheses may both have validity but at different scales of cerebral organization and function. A greater degree of asymmetry within the left hemisphere may allow for more efficient phonological processing, perhaps due to greater hemispheric specialization [97]. People at risk for impaired phonological processing may have relatively preserved function due to asymmetric development of brain regions that serve performance monitoring or other domain general functions [98] to optimize performance.

## Methods

### Participants

Retrospective data from 424 children (mean age = 10.54 years, range 6.19 to 16.92; 39% female) across 10 research sites were included in this study. In addition, retrospective data from 300 adults (mean age = 20.86 years, range 18.00 to 25.00; 53% female) across 8 research sites were included in this study. These sites included sampling approaches that targeted cases with reading disability from clinical settings, schools specializing in dyslexia remediation, as well as recruitment of participants from the local community. That is, data included in the current study were from reading disability studies and individual difference studies of reading development. Integrating these data provided pediatric and adult samples with normal distributions in phonological decoding, real word reading, verbal comprehension, and reading comprehension (Table D in S1 Appendix). Human subject research approvals and written informed consent for the original research were obtained at each institution, and the data were deidentified prior to sharing; thus, the dataset is considered nonhuman subject data. Data sharing for these research data was approved by the contributing institutions and the Medical University of South Carolina Institutional Review Board (protocol # 20606), and the research was performed according to the principles of the Declaration of Helsinki. A subset of this data has been studied previously to show the application of methods for multisite retrospective data and persistent homology analysis of structural asymmetry data [10,61,99–101].

The 2 English-speaking samples allowed for replication of results across samples with different age distributions, included children old enough to perform the reading-related tasks, and limited the influence of neurodegenerative effects of aging on the structural asymmetries [102]. Inclusion criteria also included limited artifact based on visual inspection of the T1-weighted images and limited missing data (missingness is described further in the Statistics section). In addition, a case–control propensity score matching approach was used so that age, sex, research site, and total brain volume (summed SPM12 gray matter and white matter segmented images) would be balanced across the distribution of reading skill measures that were used to profile typical and atypical reading skill development [103]. This approach was motivated by observations in a previous study that verbal comprehension was related to total brain volume [100], and, thus, we sought to limit total brain volume effects on the structural asymmetry results, as well as potential effects of age, sex, and research site.

### Imaging data and image processing (Fig 1A–1C)

T1-weighted images (Table A in S1 Appendix) were denoised [104], bias field corrected using the SPM nonuniformity (bias/apply) functions, and rigidly aligned to the MNI 152 T1 1mm template using the SPM coregistration function. Study-specific templates for the pediatric sample and adult sample were then created with the processed images and left–right flipped copies using ANTs (v 2.0) [105], as described previously [59].

The symmetrical templates were used as targets for a series of 3 normalization iterations that were increasingly focal in their magnitude of spatial warping [59]. This normalization procedure included the following parameters: [Normalization parameters- Iteration 1 (50 × 1 × 1): Cross-correlation metric (mm radius = 4.00), SyN (1.00), Gaussian regularization (3.00); Iteration 2 (1 × 50 × 1): Cross-correlation metric (mm radius = 2.00), SyN (0.75), Gaussian regularization (2.00); Iteration 3 (1 × 1 x× 40): Cross-correlation metric (mm radius = 1.00), SyN (0.50), Gaussian regularization (1.00)]. ANTS normalization was also used to spatially normalize the adult symmetrical template to the pediatric symmetrical template so that the normalization warps could be applied to the adult asymmetry images. This approach allowed for an appropriate warping of the adult images to a common coordinate space and use of the same ROI [60] in both samples.

The ANTS-derived normalization parameters from each of the 3 normalization iterations were combined to generate warps that described how to align each native space image to the symmetrical template. The ANTS Jacobian function [106] was used to produce a log-scaled Jacobian determinant image from the normalization warps that summarized the volumetric expansion and contraction necessary for aligning voxels in each image to corresponding voxels the respective pediatric or adult template (Fig 1B). Jacobian determinant values were higher for brain regions that had to be reduced in size to fit to the template and lower for brain regions that had to be increased in size to fit to the template. An 8-mm Gaussian kernel was used to smooth the images for consistency with previous voxel-based studies [107,108]. Each smoothed Jacobian image was then subtracted by its left–right flipped copy to create a hemispheric asymmetry image. That is, spatially homologous voxels between the hemispheres were subtracted to create Jacobian asymmetry images (Fig 1C). These asymmetry images produce group-level asymmetry effects that are similar to modulated gray matter volume and surface area asymmetries [25,36,109,110]. Here, the children and adults exhibited a very similar pattern of structural asymmetries (Fig F in S1 Appendix), but structural asymmetries were not directly compared between samples because age group and research site were confounded. That is, there was no way to control for site effects on image contrast that could influence age-group differences.

### Multimodal-defined regions of interest (Fig 1D)

Structural asymmetries were examined within left hemisphere ROI and right hemisphere ROI that were defined using multimodal imaging data where distinct structural and functional regions overlap across Human Connectome Project (HCP) participants. This HCP multimodal parcellation (HCP-MMP1.0) was based on task-based activity patterns, resting state connectivity between regions, and estimates of myelin density [60]. The original surface parcellations were converted to image volumes (https://doi.org/10.6084/m9.figshare.3501911.v5) and used to collect data within the space of each ROI, as in [111,112], which had been warped into the space of the pediatric template using the ANTs normalization procedure described earlier. Here, the MNI2009a T1-weighted image was normalized to the pediatric symmetric template, and the normalization warps were then applied to the gray matter ROI. Thus, the ROI are thus spatial approximations of the surface parcellations (Fig 1D).

### Persistent homology (Fig 1E and 1F)

**Fig 4:**
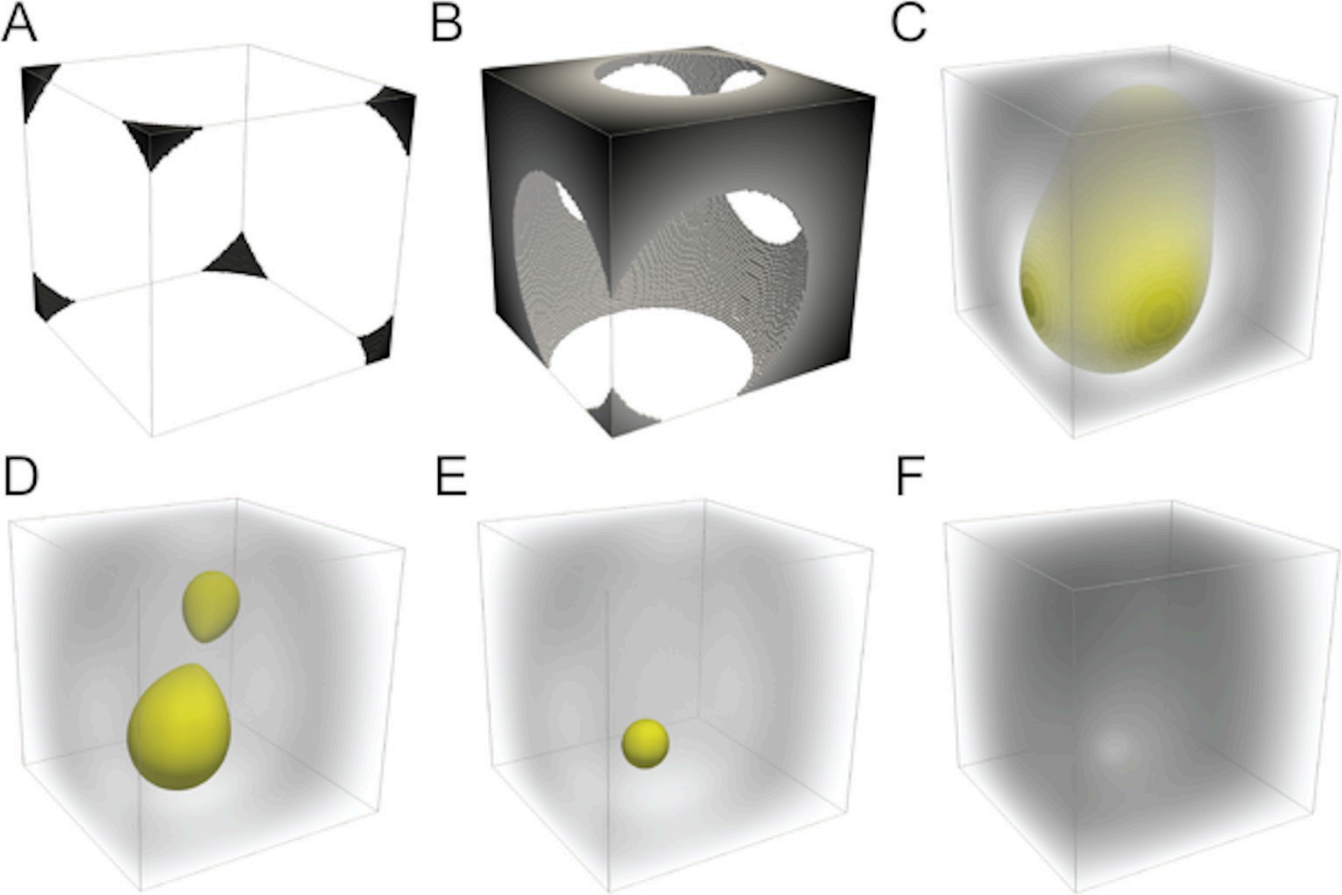
Example of the sublevel set filtration defined on a 3D image using synthetic data. Yellow surfaces indicate cavities appearing and disappearing in the filtration from (**A**–**F**). **(A)** Low-intensity voxels are introduced first, generating independent components (black regions). **(B)** While the object grows, these components merge together. **(C)** Eventually, all voxels on the boundary of the image are introduced, thus creating a cavity, with the border depicted in yellow. **(D)** With an increasing intensity threshold, the cavity splits into 2 objects. **(E)** The newly born cavity is filled and has a relatively short life span because it was born with a relatively high value that defined its boundary. Thus, it would be represented at the upper right of a persistence diagram (e.g., Fig 5). **(F)** The last and most hyperintense cavity is filled up once all voxels across intensity values are introduced.

Persistent homology is a widely used approach, rooted in topological data analysis, for analyzing shape features by means of a filtration [113]. For an imaging study like this one, filtration can be defined as the sequence of sublevel sets of image signal intensity. In this study, filtration involves growing an object by introducing voxels according to their signal intensity from hypointense to hyperintense voxel values, as shown in Fig 4.

**Fig 5:**
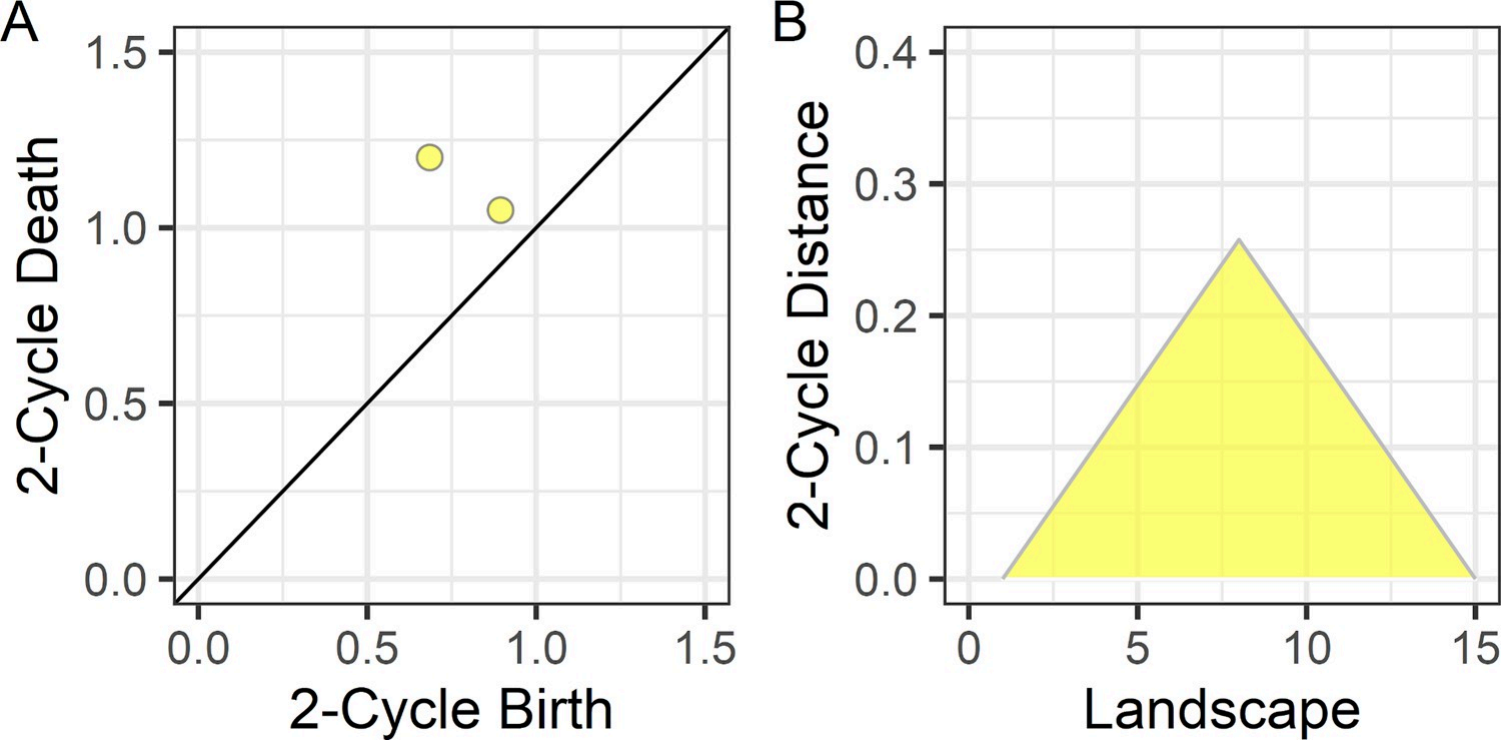
Persistence diagram and landscape examples of the image shown in Fig 4. **(A)** Two persistence pairs or 3D structures were identified in the synthetic data shown in Fig 4. **(B)** The persistence landscape represents the persistence pair with the maximum distance in the persistence diagram across the range of birth values or low intensity boundary for each object.

Filtration by increasing signal intensity of the asymmetry image forms topological features that are described and quantified by persistent homology. Here, the range of signal intensity of a topological feature or asymmetric structure was described by a pair of indices or a persistence pair. This persistence pair describes when in the filtration the topological feature was generated (birth) and destroyed (death). In this work, we focused on 2-cycles, which are 3D hyperintense objects in the asymmetry images that are surrounded by relatively hypointense voxel values. In the simplest case where a single cluster of hyperintense voxel values was surrounded by hypointense voxels, the birth of the object would be the smallest voxel value where a 2-cycle was identified or the local minima boundary of the structure, and the death of the object would be the most hyperintense voxel value of that object or its highest voxel value. In Fig 4, 2-cycles correspond to the yellow surfaces appearing and disappearing in the filtration. Each time a 2-cycle appears for the first time (e.g., Fig 4C), we say a 2-cycle was born. When the 2-cycle disappears (e.g., Fig 4E), we say a 2-cycle died. The collection of persistence pairs is commonly represented by a persistence diagram (Figs 1E and 5A). The Topology Toolkit (v.0.9.8; [114]) and custom scripts available at the Open Science Framework URL below were used to compute persistence pairs from each participant’s asymmetry images. For each ROI, persistence pairs were included in the corresponding persistence diagram only if the peak voxel value (or death of the asymmetry) occurred within the ROI.

Persistence landscapes were used to represent the persistence diagrams as a normalized vector space that quantifies the life span (that is, minimum intensity birth or boundary to the maximum intensity death or peak value of the structure; Figs 1F and 5B) of asymmetries across the landscape or range of asymmetry values [115]. This transformation of the persistence diagram into a landscape allowed for statistical comparisons at each landscape position across participants who can have a different number of persistence pairs. The R TDA library (v.1.6.5) was used to create persistence landscapes that represented persistence pairs across 15 positions across the range of asymmetry values in the images (Figs 1F and 5B), where there is no data in the first and last position (that is, analyses were performed at 13 landscape positions) [61].

### Image analysis approach comparisons

A priori and data driven approaches commonly used for reducing the dimensionality of neuroimaging data include averaging values within ROI and group ICAs, respectively. For comparison of results to the persistent homology approach, we averaged asymmetry values within ROI that exhibited significant quadratic associations between the persistent homology and phonological decoding measures. These average values were then correlated with phonological decoding performance using the same approach that was used for the persistent homology data.

Group ICA was also performed with the pediatric and the adult data using the Group ICA of fMRI Toolbox (GIFT; v.3.0b) to reduce the dimensionality of the data to weights or values that represented each participant’s contribution to unique patterns of spatially covarying Jacobian asymmetries. Participants with a larger component weight had a more exaggerated asymmetry for that component. GIFT is commonly used for ICA of fMRI data and has been used for structural analyses, including in our laboratory (e.g., 7 gray matter components in a cross-sectional sample of younger to older adults [116]). The GIFT analyses demonstrated only 2 components in the pediatric and adult samples, which had similar spatial distributions in both samples (Fig E in S1 Appendix) and occurred where there were significant asymmetries across participants in each sample (Fig F in S1 Appendix). However, these components accounted for just 5.29% and 6.07% of the variance, respectively. That is, the ICA approach characterized patterns of significant asymmetries that were common across participants, but there was considerable individual variability in the covariance structure of Jacobian asymmetries across brain regions.

### Statistical analyses

R (v 3.6.0) was used for statistical analyses. This included the use of the Multivariate Imputation by Chained Equations (MICE) library (v 3.6.0) to perform multiple imputation for dealing with behavioral measure missingness [100,117]. Multiple imputation was used to create 10 imputed datasets from a sample of 1,576 participants in the larger Dyslexia Data Consortium. This larger sample exhibited 10% missingness across predictor variables in the multiple imputation model, which included age, sex, research site, as well as standardized scores for phonological decoding (Word Attack [118,119]), real word identification (Letter–Word Identification [118,119]), reading comprehension (Passage Comprehension cloze task [118,119]), rapid naming [120,121], and verbal comprehension [122,123]. These measures were requested during multisite data collection, in part because they have been used in previous studies to demonstrate brain structure and behavior associations, particularly for the Word Attack phonological decoding measure [9,39,41,72,124,125]. There was 6% and 14% missingness in the pediatric and adult samples, with a low percentage of phonological decoding missingness (0.4%, 8%), respectively. Table D in S1 Appendix shows the pooled Pearson correlation coefficients for each pair of behavioral variables across the 10 imputed datasets.

Linear and quantile regression analyses were performed with the 10 multiply-imputed datasets for each landscape position representing asymmetries across all left or right hemisphere ROI, as well as within ROI, to test the cerebral lateralization hypothesis that more exaggerated asymmetries confer a phonological processing advantage, and the canalization hypothesis that more exaggerated asymmetries constrain the phonological processing to the average range. That is, asymmetries were predicted to have a linear association with phonological processing, as examined with linear regression, according to the cerebral asymmetry hypothesis; and a nonlinear inverted-U sandpile association, as examined with quantile regression, according to the canalization hypothesis. Age, sex, and research site control variables were regressed to examine asymmetry and phonologic decoding effects that were independent of these variables. Random noise (−0.00001 to 0.00001) was added to the vector of participant asymmetry data at each landscape position because of the potential for convergence problems when many zero values are included in quantile regressions. For consistency, the same random noise approach was used for the linear regression analyses. Similar linear regression results were observed without using this noise approach.

The cerebral lateralization hypothesis was examined using standard linear regressions for the landscape data that was aggregated across left or right hemisphere ROI. The canalization hypothesis was examined using quantile regression (quantreg library (v.5.75) [126]) because the landscape data for each ROI exhibited a Weibull or leftward skewed distribution, as seen in the ROI scatter plots (Fig 3, Fig I in S1 Appendix). In contrast, landscape data aggregated across the left or right hemisphere had a more normal distribution and were appropriately examined using linear regression. In addition, quantile regression allowed for the analysis of data at the 99th percentile (tau = 0.99) to examine quadratic effects for an inverted-U sandpile shape of the data as evidence for canalization. For all quantile regressions, a second order polynomial term was used to examine orthogonal linear and quadratic associations.

Bootstrap samples (10,000) were generated for each analysis using the boot library (v.1-3.22) to control for outlier values. In addition, children and adults with outlier Word Attack standard scores (children: 47 and 55; adults: 150) were excluded from the linear and quantile regressions to limit extreme values from influencing the results (Children: multiply imputed Word Attack mean = 97.74, sd = 14.62; Adults: multiply imputed Word Attack mean = 99.97, sd = 10.10). Finally, the results for each landscape position were averaged across the 10,000 bootstrap and 10 imputation datasets. That is, results from 100,000 statistical tests were pooled for each landscape position to ensure stability of the results and validity of inference.

### Statistical testing

A review of the literature indicated that the association between planum temporale asymmetry and reading disability has a relatively small effect size [33], which may require large samples to observe a significant result with sufficient statistical power using a *p* < 0.05 threshold. Thus, small effect sizes were anticipated in this study, and this is one reason for the use of a replication sample and bootstrap analyses. That is, effects were considered significant (*p* < 0.05) only if they occurred in both relatively large samples.

Persistence landscape values at adjacent positions can be highly correlated and thus exhibit a spatial dependency and statistical nonindependence. For this reason, the persistence landscape statistical results for each ROI were corrected for multiple comparisons with a permutation approach to establish extent-based thresholds for effects that spanned multiple adjacent landscape positions [127]. After positive or negative statistical results were thresholded based on an uncorrected two-tailed alpha < 0.05, each landscape position was randomly reordered with replacement for each 10,000 times within-ROI to generate a null distribution for the frequency of adjacent significant results across landscapes. This approach quantified the frequency and number of adjacent position effects that would occur by chance (that is, without an underlying structure producing adjacent results), to estimate the probability of multiple adjacent effects across landscape positions. Results were then counted across landscapes to determine the 95% cutoff for the number of adjacent landscape position effects. By defining significant effects with less than a 5% likelihood of occurring randomly, the procedure provided an empirically based family-wise error correction that accounted for spatial dependencies in the observed data. Together, the statistic testing methods ensured that significant effects were consistent across samples and across persistence landscapes and were not dependent on spatially isolated outlier values.

### Code, data availability, and computational resources

The code for generating asymmetry images using T1-weighted images, as described in [59], is available at the following URL: https://www.nitrc.org/projects/structural_asym. The asymmetry images, symmetrical brain template, ROI, behavioral and demographic data, and persistent pairs from each ROI, and data analysis code used in this study are accessible through https://osf.io/75g9d. Finally, the statistical analyses used to test the cerebral lateralization hypothesis were performed using a standard 290 GHz laptop with 16 GB of RAM and a Windows 10 operating system. The statistical analyses used to test the canalization hypothesis were performed using the Clemson Palmetto cluster to speed the processing time for the many analyses performed across bootstraps, imputations, landscapes positions, and ROI. We recommend focusing on specific ROI (that is, the dorsal paracingulate) to replicate the canalization results using the code at the OSF URL above.

## Supporting information

S1 Appendix Underlying data and code: https://osf.io/75g9d (see code_data_for_suppFigs_2_3_7.zip in the results/figures directory). **Fig A.** The persistent homology measure of structural asymmetry in BA 8bm was associated with the extent of voxel-based asymmetries in the same region, as validation that the persistent homology measure of asymmetry represents the underlying voxel values. In addition to the effects in BA 8bm (*p* < 0.05 FWE), children (dark blue) and adults (light blue) also exhibited spatially overlapping associations (red) in the right anterior cingulate and forceps minor of the corpus callosum (*p* < 0.001 uncorrected). These voxel-based correlation analyses were performed with the y-axis data presented in Fig 3B, while controlling for age, sex, and research site. Images used to generate this image: https://osf.io/75g9d.
**Fig B**. Positive linear associations between persistence landscape data from each ROI and phonological decoding. No significant effects were observed in pediatric or adult samples. Thus, these results provided limited support for the cerebral lateralization hypothesis at the spatial scale of a locally specific ROI. That is, FWE corrected effects were observed only for the children (dark red), including the right temporal parietal junction (TPOJ2) and inferior frontal and parietal gyri (43, 44), but similar effects were not observed in the adults. These analyses were performed for each landscape position (2–14) using 10,000 bootstrap samples and results were averaged across 10 multiply imputed datasets. Labels for the Glasser ROI: https://bitbucket.org/dpat/tools/src/master/REF/ATLASES/Glasser_2016_Table.xlsx.
**Fig C**. Negative linear associations between persistence landscape data from each ROI and phonological decoding. No significant effects were observed across the pediatric and adult samples. Thus, these results provided limited support for the cerebral lateralization hypothesis at the spatial scale of a locally specific ROI. That is, FWE corrected effects were observed only for the children (dark blue), including the right PBelt region that is part of the planum temporale, but similar effects were not observed in the adults. These analyses were performed for each landscape position using 10,000 bootstrap samples, and results were averaged across 10 multiply imputed datasets. Labels for the Glasser ROI: https://bitbucket.org/dpat/tools/src/master/REF/ATLASES/Glasser_2016_Table.xlsx.
**Fig D**. Individual variation in the most asymmetric structure across the left hemisphere ROI, but not the right hemisphere ROI, exhibited significant associations with phonological decoding. The left hemisphere results are the same that are shown in Fig 2A and 2B, but the plot axes are rotated to show the nonsignificant right hemisphere effects across adjacent left hemisphere landscape positions. The pink bar located at the right landscape position 3 in A represents a negative association (*p* < 0.05). As in Fig 2, red bars indicate *p* < 0.05 associations that were present in both children and adults for that landscape position, whereas the pink bars indicate *p* < 0.05 associations that were present only in one sample.
**Fig E**. Overlapping spatial patterns of voxel-based Jacobian asymmetries with common covariance structure were observed in children and adults but were not significantly associated with phonological decoding. (**A)** Group ICA demonstrated an independent component that was represented by left occipital and left cerebellar hemisphere asymmetries in children (dark blue) and adults (light blue), with considerable spatial overlap between the samples (red). (**B)** An independent component was also represented in both samples by left Heschl’s gyrus, anterior insula/medial inferior frontal gyrus, parietal cortex, and hippocampus (rightward effect for the superior temporal sulcus and parieto-occipital sulcus) asymmetries. A similar spatial distribution of effects is shown in Fig E in S1 Appendix for significant one-sample *t* test asymmetry results. (**C, D)** Phonological decoding (Word Attack: WA-SS) was not significantly related to the weights representing how much each participant contributed to each independent component. IC1: the occipital/cerebellar component shown in (**A)**. IC2: the medial Heschl’s gyrus/planum temporal component shown in (**B)**. Images shown in (A) and underlying data and code for (C) and (D): https://osf.io/75g9d.
**Fig F**. One-sample *t* test results showing significant voxel-based asymmetries (*p* < 0.05 FWE) in children (dark blue) and adults (light blue), with considerable spatial overlap of results across samples (red). These results demonstrate that structural asymmetries based on Jacobian determinant estimates of volumetric expansion and contraction required for normalization to the study specific template exhibited spatial patterns of asymmetry that have been reported in studies examining voxel-based gray matter and surface area [25,109,110]. Direct comparisons of the pediatric and adult asymmetry data were not performed because of limited overlap of research sites across pediatric and adult datasets. Images: https://osf.io/75g9d.
**Fig G**. No consistent evidence for canalization was observed when phonological decoding was correlated with individual differences in the maximum structural asymmetry across the left or right hemisphere ROI for the children (**A)** or adults (**B)**. The quadratic quantile (99th percentile) regression analyses demonstrated inconsistent and nonsignificant inverted-U associations between structural asymmetries across the left or right hemispheres and phonological decoding (Word Attack). The landscapes are presented vertically to show left and right hemisphere effects. Cohen’s *d* values in (**A)** and (**B)** are presented as absolute values. Only the right landscape position 10 for the adults exhibited an inverted U (negative) association with phonological decoding.
**Fig H**. Inverted-U quadratic associations between the structural asymmetry topology data from each ROI and phonological decoding (Word Attack). Family-wise error corrected results in both samples (yellow) provided support for the canalization hypothesis that structural asymmetries constrain phonological decoding within the normal range, as demonstrated by significant effects in left premotor (6D), left dorsal cingulate (8bm), right subgenual cingulate (25), and right lateral occipital (LO1) ROI. FWE corrected effects were observed only for children (dark blue) and only for adults (light blue) but were not considered significant effects because they did not replicate across samples. These analyses were performed for each landscape position using 10,000 bootstrap samples and results were averaged across 10 multiply imputed datasets. Labels for the Glasser ROI: https://bitbucket.org/dpat/tools/src/master/REF/ATLASES/Glasser_2016_Table.xlsx.
**Fig I.** Dorsal cingulate/paracingulate asymmetry associations with real word decoding/reading, passage comprehension, verbal comprehension (Verbal IQ), and rapid automatized naming. These associations exhibited varying effect sizes but were large enough that the asymmetry association with phonological decoding was no longer significant after controlling the variance from the other behavioral measures in phonological decoding.
**Fig J.** Participants with the most leftward asymmetries in the BA 8bm ROI had a cingulate sulcus (blue line) and a paracingulate sulcus (red line). (**A)** Template image with the BA 8bm ROI overlaid (red). (**B)** Row of adult images with BA 8bm leftward asymmetries at the 99th percentile. (**C)** Row of children with BA 8bm leftward asymmetries at the 99th percentile. Note the parallel labeled sulci, which are largely continuous in the children.
**Table A.** T1-weighted image parameters from the study sites.
**Table B.** Significant linear associations between leftward structural asymmetries across the left cerebral hemisphere and phonological decoding (in bold) were not substantively affected after controlling for total brain volume (summed total gray matter and white matter volume).
**Table C.** Linear associations between reading-related measures and left hemisphere persistence distance for the landscape position where the phonological decoding measure was maximally associated with structural asymmetries (Children: Position 7; Adults: Position 10).
**Table D.** Descriptive statistics and Pearson correlations for the reading-related variables in the pediatric and adult samples.
**Table E.** Significant inverted-U associations between BA 8bm or BA 6d leftward structural asymmetries and phonological decoding (in bold font) were largely unaffected after controlling for total brain volume (summed total gray matter and white matter volume). BA, Brodmann area; FWE, family-wise error; ROI, region of interest.
(DOCX)

## Abbreviations

HCP: Human Connectome Project
ICA: independent component analysis
MICE: Multivariate Imputation by Chained Equations
ROI: region of interest
