# Supplementary material for: Cortical asymmetries at different spatial hierarchies relate to phonological processing ability
Source: PLoS Biol. 2022 Apr 5;20(4):e3001591. doi: 10.1371/journal.pbio.3001591 (PMC8982829; doi:10.1371/journal.pbio.3001591)
Supplement: S1 Appendix — Fig A. The persistent homology measure of structural asymmetry in BA 8bm was associated with the extent of voxel-based asymmetries in the same region, as validation that the persistent homology measure of asymmetry represents the underlying voxel values. In addition to the effects in BA 8bm (p < 0.05 FWE), children (dark blue) and adults (light blue) also exhibited spatially overlapping associations (red) in the right anterior cingulate and forceps minor of the corpus callosum (p < 0.001 uncorrected). These voxel-based correlation analyses were performed with the y-axis data presented in Fig 3B, while controlling for age, sex, and research site. Images used to generate this image: https://osf.io/75g9d. Fig B. Positive linear associations between persistence landscape data from each ROI and phonological decoding. No significant effects were observed in pediatric or adult samples. Thus, these results provided limited support for the cerebral lateralization hypothesis at the spatial scale of a locally specific ROI. That is, FWE corrected effects were observed only for the children (dark red), including the right temporal parietal junction (TPOJ2) and inferior frontal and parietal gyri (43, 44), but similar effects were not observed in the adults. These analyses were performed for each landscape position (2–14) using 10,000 bootstrap samples and results were averaged across 10 multiply imputed datasets. Labels for the Glasser ROI: https://bitbucket.org/dpat/tools/src/master/REF/ATLASES/Glasser_2016_Table.xlsx. Fig C. Negative linear associations between persistence landscape data from each ROI and phonological decoding. No significant effects were observed across the pediatric and adult samples. Thus, these results provided limited support for the cerebral lateralization hypothesis at the spatial scale of a locally specific ROI. That is, FWE corrected effects were observed only for the children (dark blue), including the right PBelt region that is part of the planum tem [file pbio.3001591.s001.docx]

**S1 Appendix**


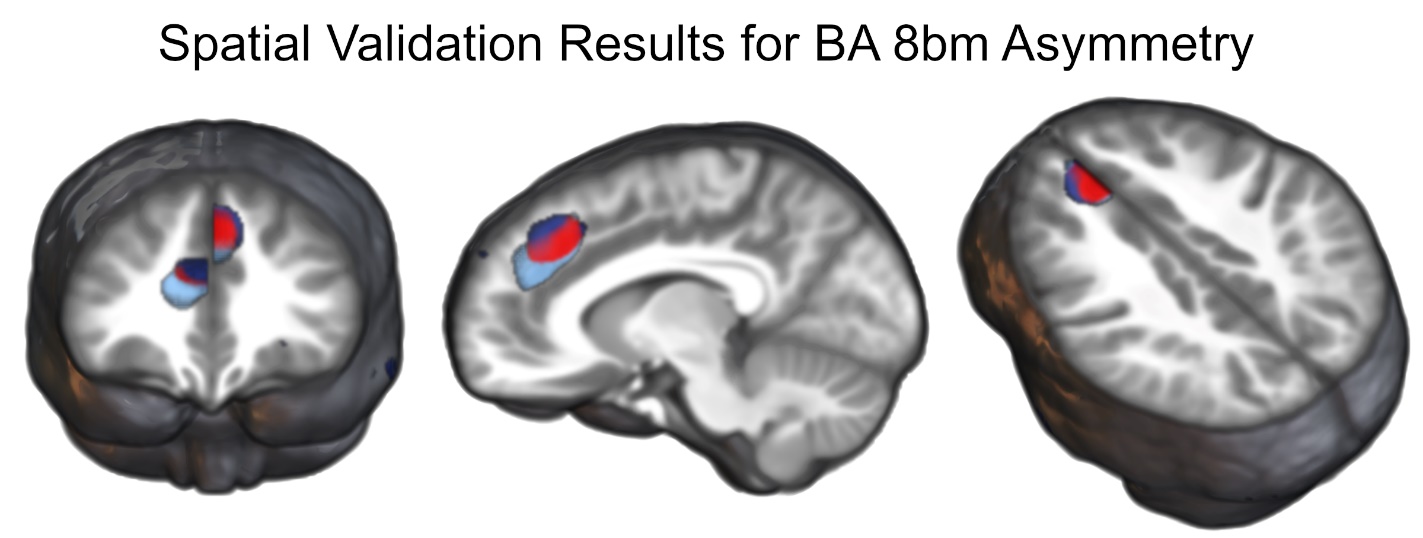


**Fig A.** The persistent homology measure of structural asymmetry in Brodmann area (BA) 8bm was associated with the extent of voxel-based asymmetries in the same region, as validation that the persistent homology measure of asymmetry represents the underlying voxel values. In addition to the effects in BA 8bm (*p* < 0.05 FWE), children (dark blue) and adults (light blue) also exhibited spatially overlapping associations (red) in the right anterior cingulate and forceps minor of the corpus callosum (*p* < 0.001 uncorrected). These voxel-based correlation analyses were performed with the y-axis data presented in Figure 3B, while controlling for age, sex, and research site. Images used to generate this image: https://osf.io/75g9d.

**
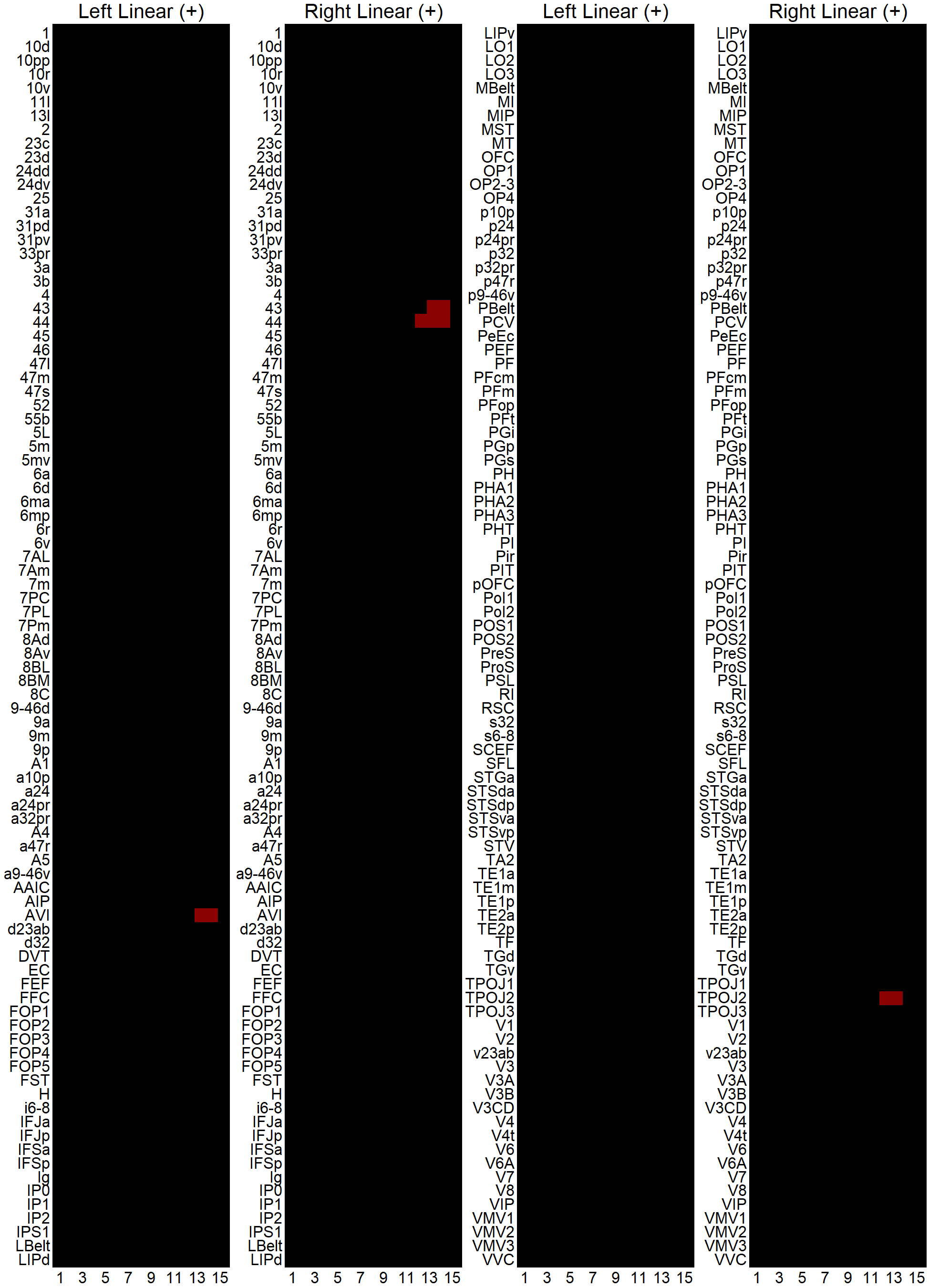
**

**Fig B**. Positive linear associations between persistence landscape data from each ROI and phonological decoding. No significant effects were observed in pediatric or adult samples. Thus, these results provided limited support for the cerebral lateralization hypothesis at the spatial scale of a locally specific ROI. That is, family-wise error corrected effects were observed only for the children (dark red), including the right temporal parietal junction (TPOJ2) and inferior frontal and parietal gyri (43, 44), but similar effects were not observed in the adults. These analyses were performed for each landscape positions (2-14) using 10,000 bootstrap samples and results were averaged across 10 multiply imputed datasets. Labels for the Glasser ROI: https://bitbucket.org/dpat/tools/src/master/REF/ATLASES/Glasser_2016_Table.xlsx

Underlying data and code: https://osf.io/75g9d (see code_data_for_suppFigs_2_3_7.zip in the results/figures directory).

**
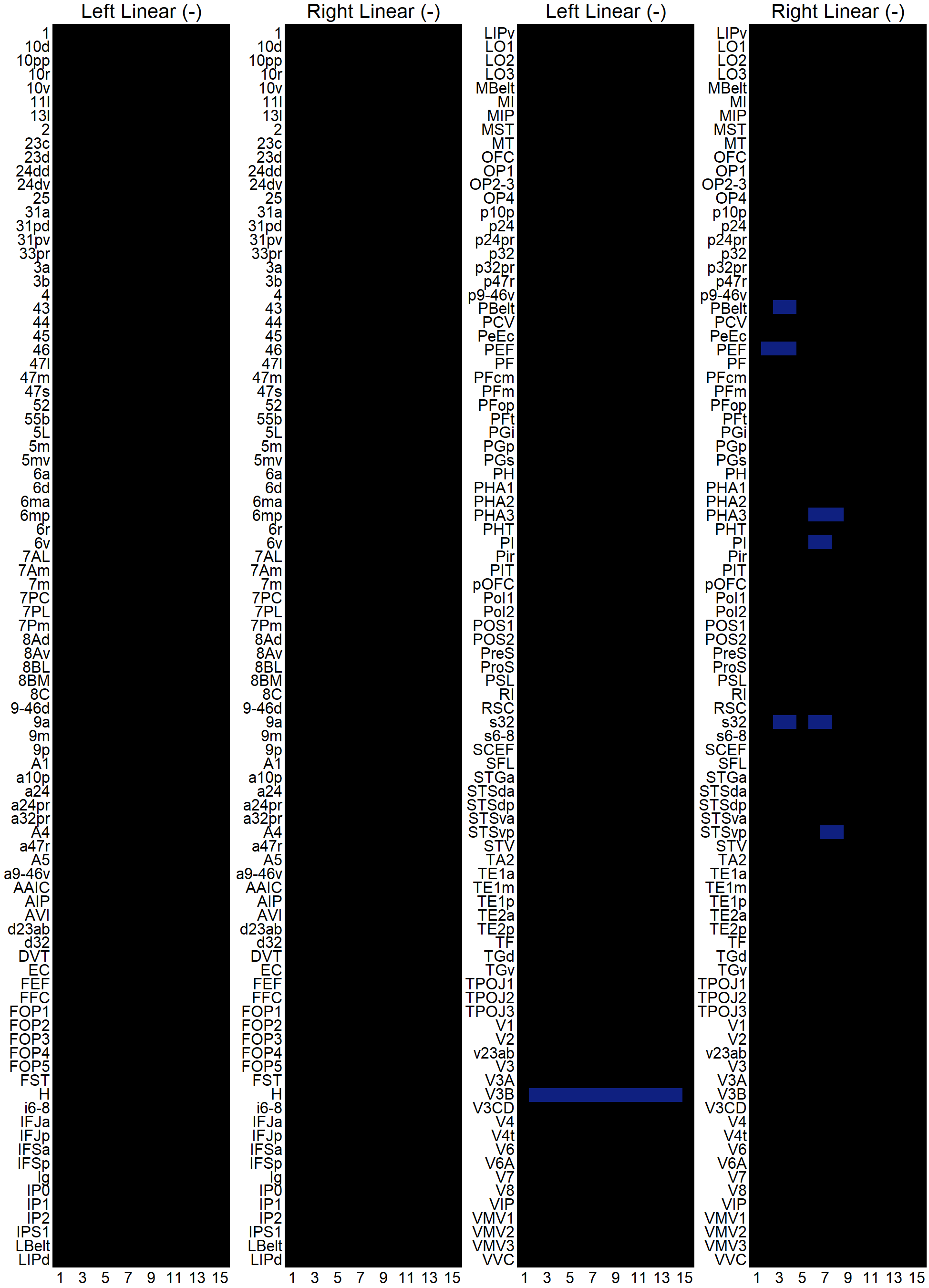
**

**Fig C**. Negative linear associations between persistence landscape data from each ROI and phonological decoding. No significant effects were observed across the pediatric and adult samples. Thus, these results provided limited support for the cerebral lateralization hypothesis at the spatial scale of a locally specific ROI. That is, family-wise error corrected effects were observed only for the children (dark blue), including the right PBelt region that is part of the planum temporale, but similar effects were not observed in the adults. These analyses were performed for each landscape position using 10,000 bootstrap samples and results were averaged across 10 multiply imputed datasets. Labels for the Glasser ROI: https://bitbucket.org/dpat/tools/src/master/REF/ATLASES/Glasser_2016_Table.xlsx

Underlying data and code: https://osf.io/75g9d (see code_data_for_suppFigs_2_3_7.zip in the results/figures directory).


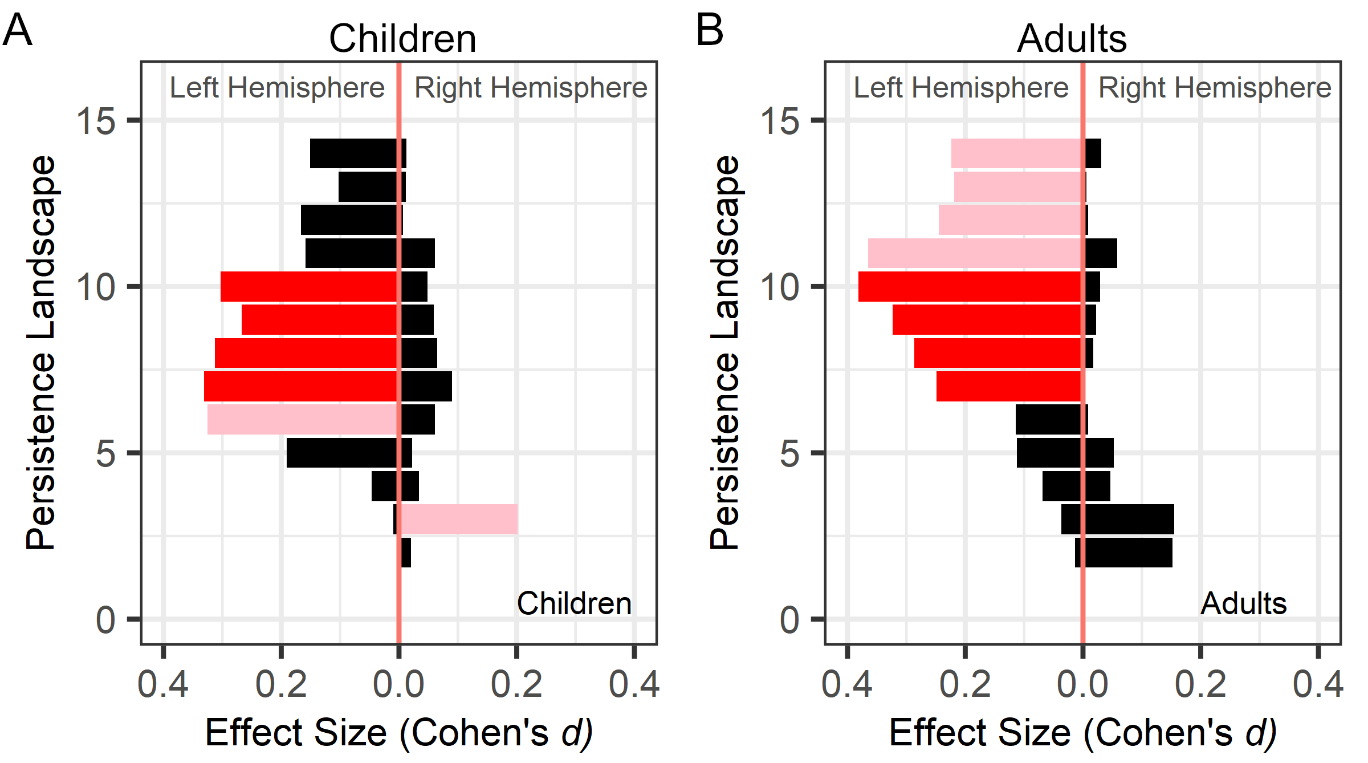


**Fig D**. Individual variation in the most asymmetric structure across the left hemisphere ROI, but not the right hemisphere ROI, exhibited significant associations with phonological decoding. The left hemisphere results are the same that are shown in Fig 2 A and B, but the plot axes are rotated to show the non-significant right hemisphere effects across adjacent left hemisphere landscape positions. The pink bar located at the right landscape position 3 in A represents a negative association (p < 0.05). As in Fig 2, red bars indicate p < 0.05 associations that were present in both children and adults for that landscape position, whereas the pink bars indicate p < 0.05 associations that were present only in one sample. Underlying data and code: https://osf.io/75g9d.

.


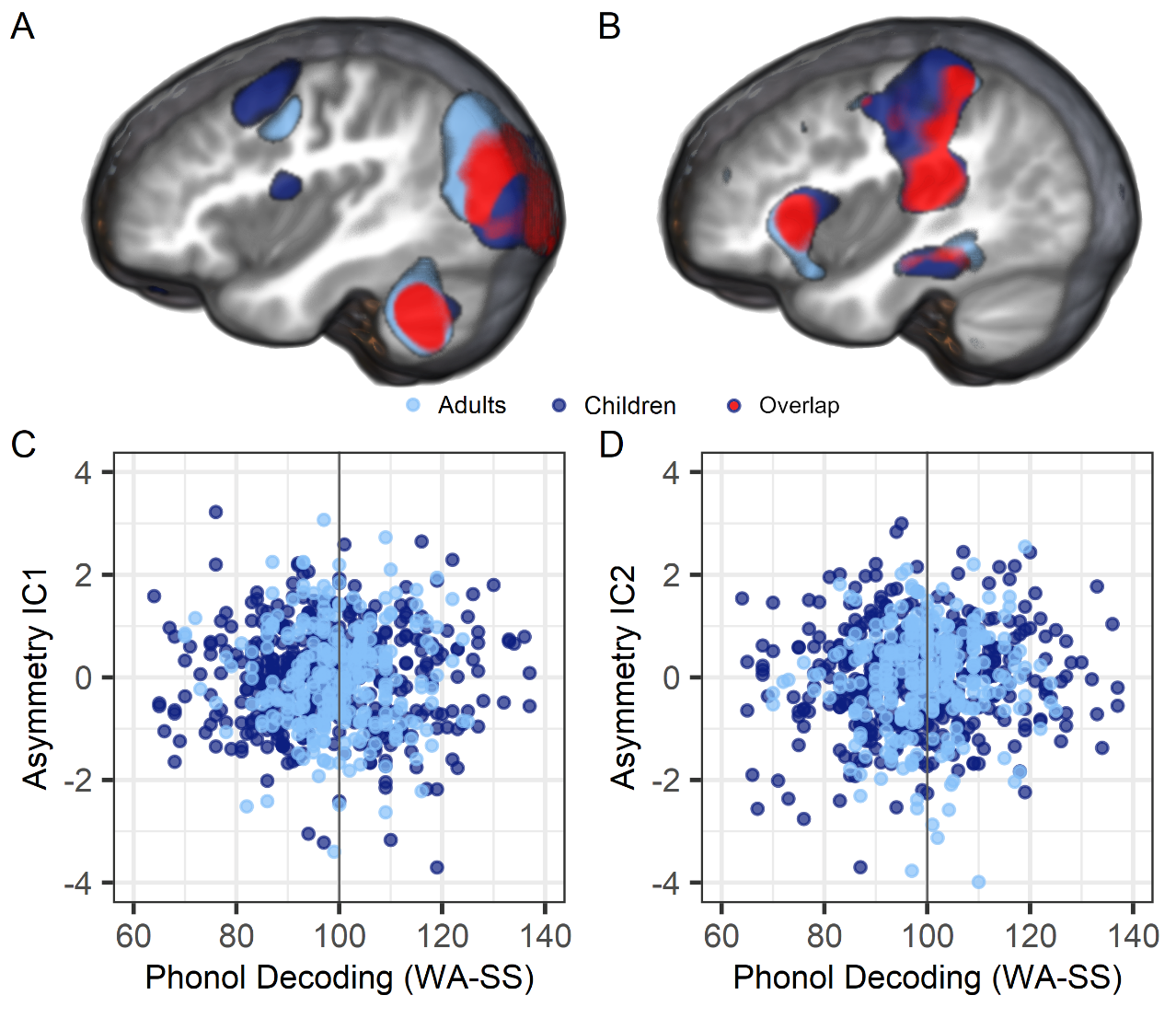


**Fig E**. Overlapping spatial patterns of voxel-based Jacobian asymmetries with common covariance structure were observed in children and adults but were not significantly associated with phonological decoding. **A**. Group independent component analysis demonstrated an independent component that was represented by left occipital and left cerebellar hemisphere asymmetries in children (dark blue) and adults (light blue), with considerable spatial overlap between the samples (red). **B**. An independent component was also represented in both samples by left Heschl’s gyrus, anterior insula/medial inferior frontal gyrus, parietal cortex, and hippocampus (rightward effect for the superior temporal sulcus and parieto-occipital sulcus) asymmetries. A similar spatial distribution of effects is shown in Supplementary Fig 5 for significant one-sample t-test asymmetry results. **C, D**. Phonological decoding (Word Attack: WA-SS) was not significantly related to the weights representing how much each participant contributed to each independent component. IC1: the occipital/cerebellar component shown in **A**. IC2: the medial Heschl’s gyrus / planum temporal component shown in **B**. Images shown in A and underlying data and code for C and D: https://osf.io/75g9d.


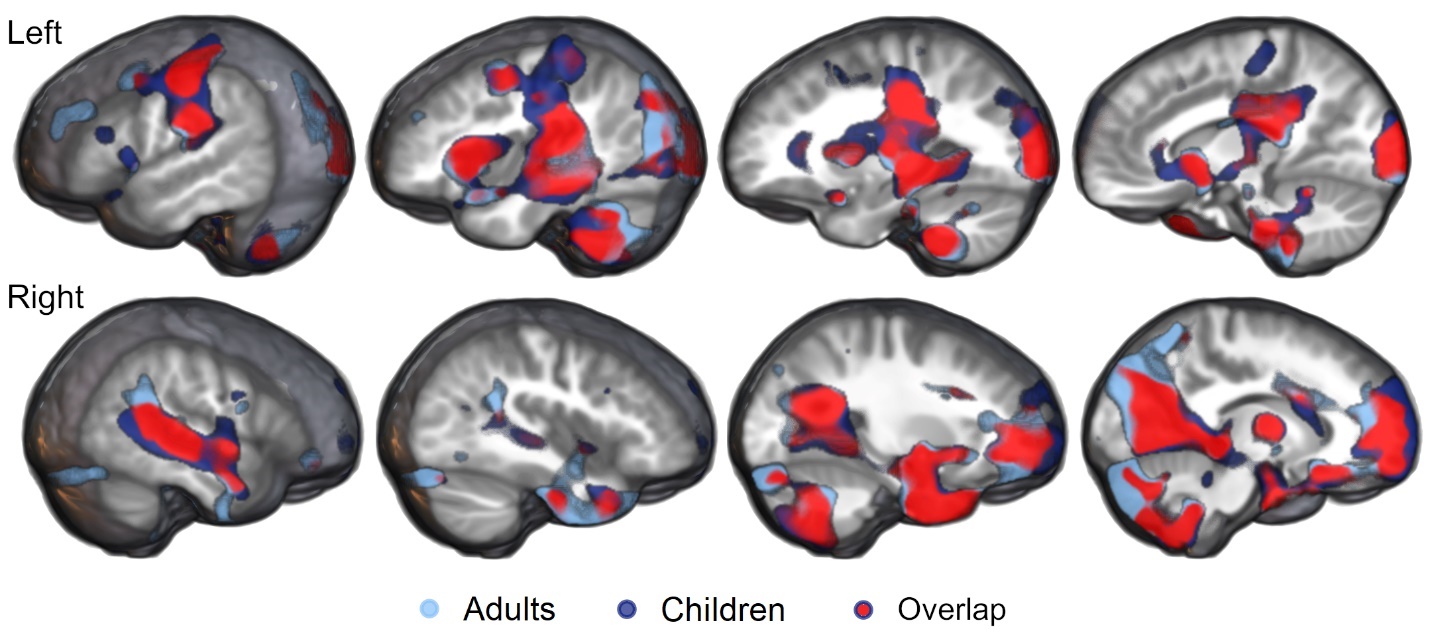


**Fig F**. One-sample t-test results showing significant voxel-based asymmetries (*p* < 0.05 FWE) in children (dark blue) and adults (light blue), with considerable spatial overlap of results across samples (red). These results demonstrate that structural asymmetries based on Jacobian determinant estimates of volumetric expansion and contraction required for normalization to the study specific template exhibit spatial patterns of asymmetry that have been reported in studies examining voxel based gray matter and surface area [25, 109, 110]. Direct comparisons of the pediatric and adult asymmetry data were not performed because of limited overlap of research sites across pediatric and adult datasets. Images: https://osf.io/75g9d.

**
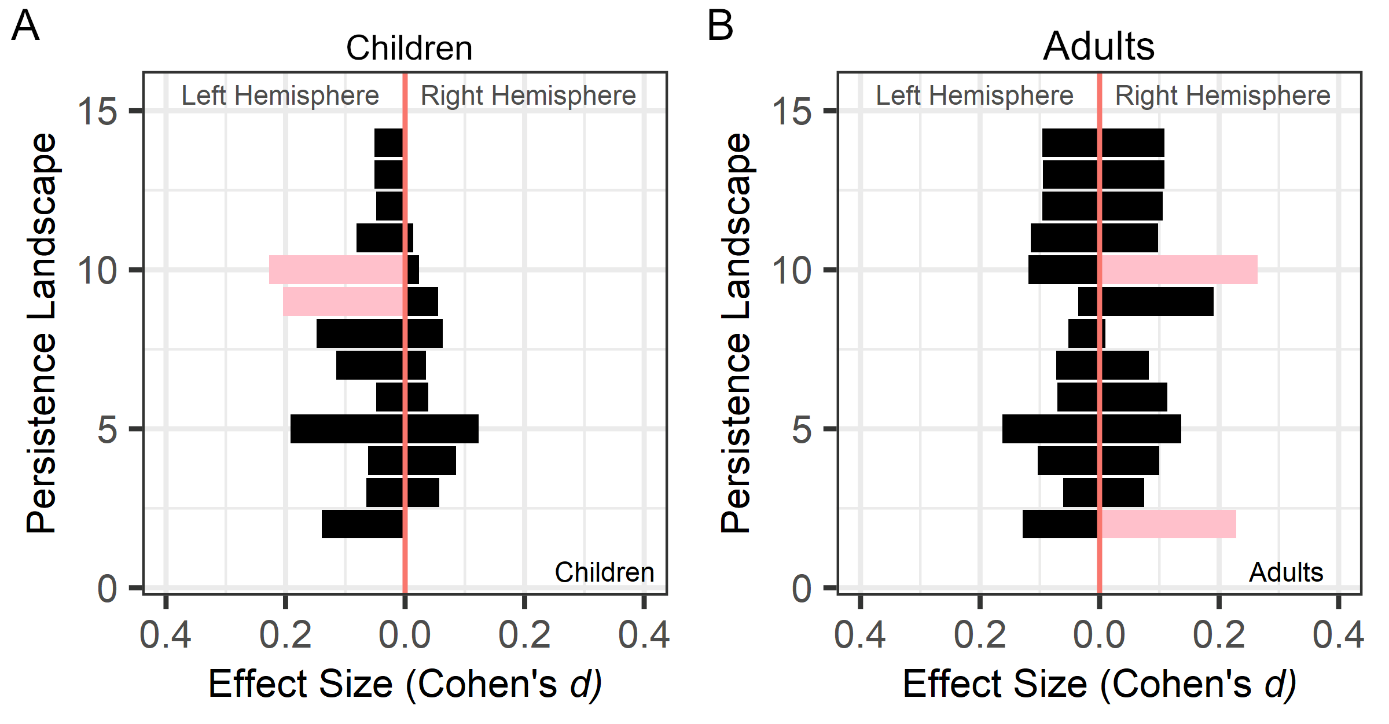
**

**Fig G**. No consistent evidence for canalization was observed when phonological decoding was correlated with individual differences in the maximum structural asymmetry across the left or right hemisphere ROI for the children **A** or adults **B**. The quadratic quantile (99^th^ percentile) regression analyses demonstrated inconsistent and non-significant inverted-U associations between structural asymmetries across the left or right hemispheres and phonological decoding (Word Attack). The landscapes are presented vertically to show left and right hemisphere effects. Cohen’s *d* values in **A** and **B** are presented as absolute values. Only the right landscape position 10 for the adults exhibited an inverted U (negative) association with phonological decoding. Underlying data and code: https://osf.io/75g9d.


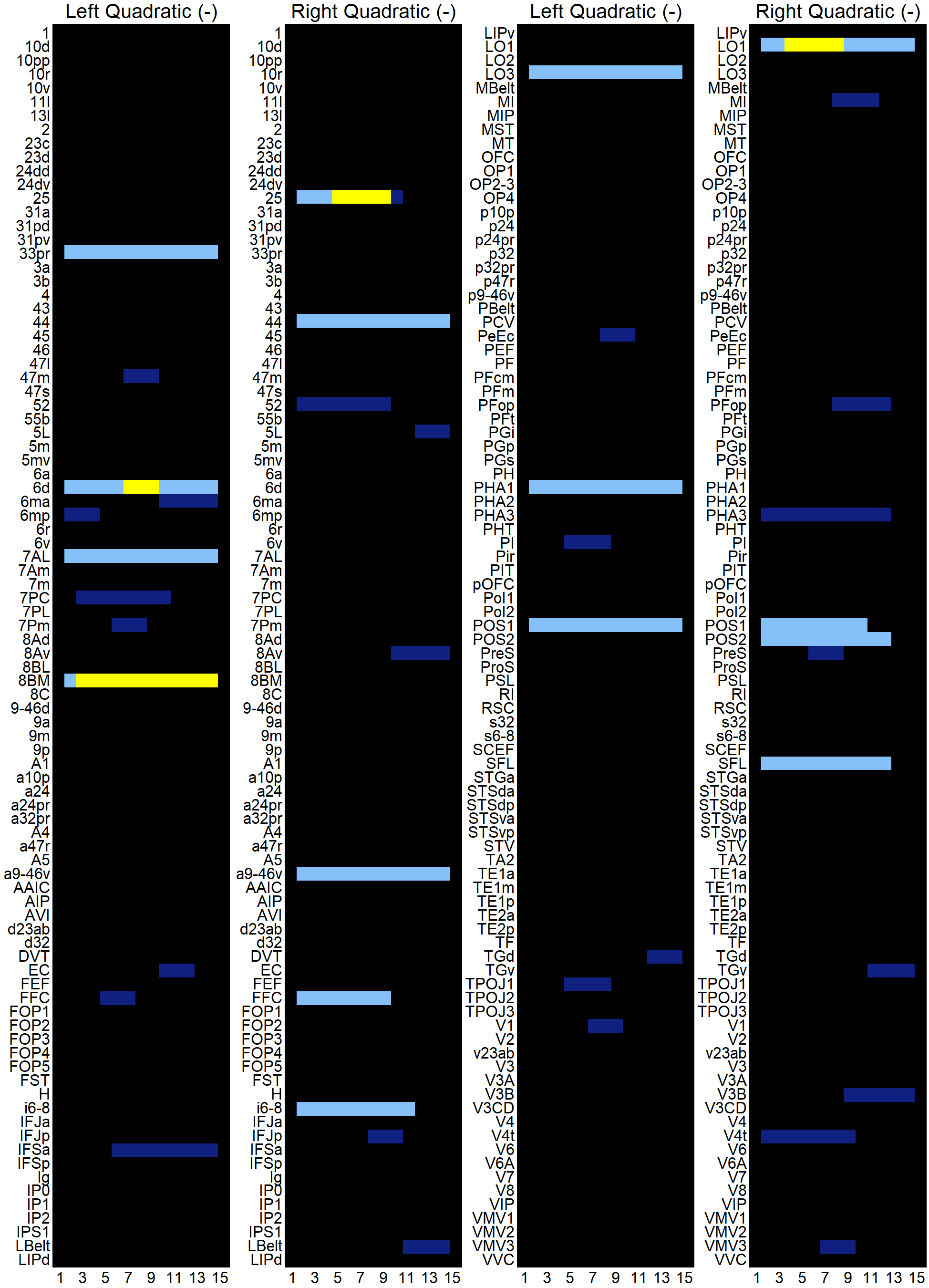


**Fig H**. Inverted-U quadratic associations between the structural asymmetry topology data from each ROI and phonological decoding (Word Attack). Family-wise error corrected results in both samples (yellow) provided support for the canalization hypothesis that structural asymmetries constrain phonological decoding within the normal range, as demonstrated by significant effects in left premotor (6D), left dorsal cingulate (8bm), right subgenual cingulate (25), and right lateral occipital (LO1) ROI. Family-wise error corrected effects were observed only for children (dark blue) and only for adults (light blue) but were not considered significant effects because they did not replicate across samples. These analyses were performed for each landscape position using 10,000 bootstrap samples and results were averaged across 10 multiply imputed datasets. Labels for the Glasser ROI: https://bitbucket.org/dpat/tools/src/master/REF/ATLASES/Glasser_2016_Table.xlsx

Underlying data and code: https://osf.io/75g9d (see code_data_for_suppFigs_2_3_7.zip in the results/figures directory).

**
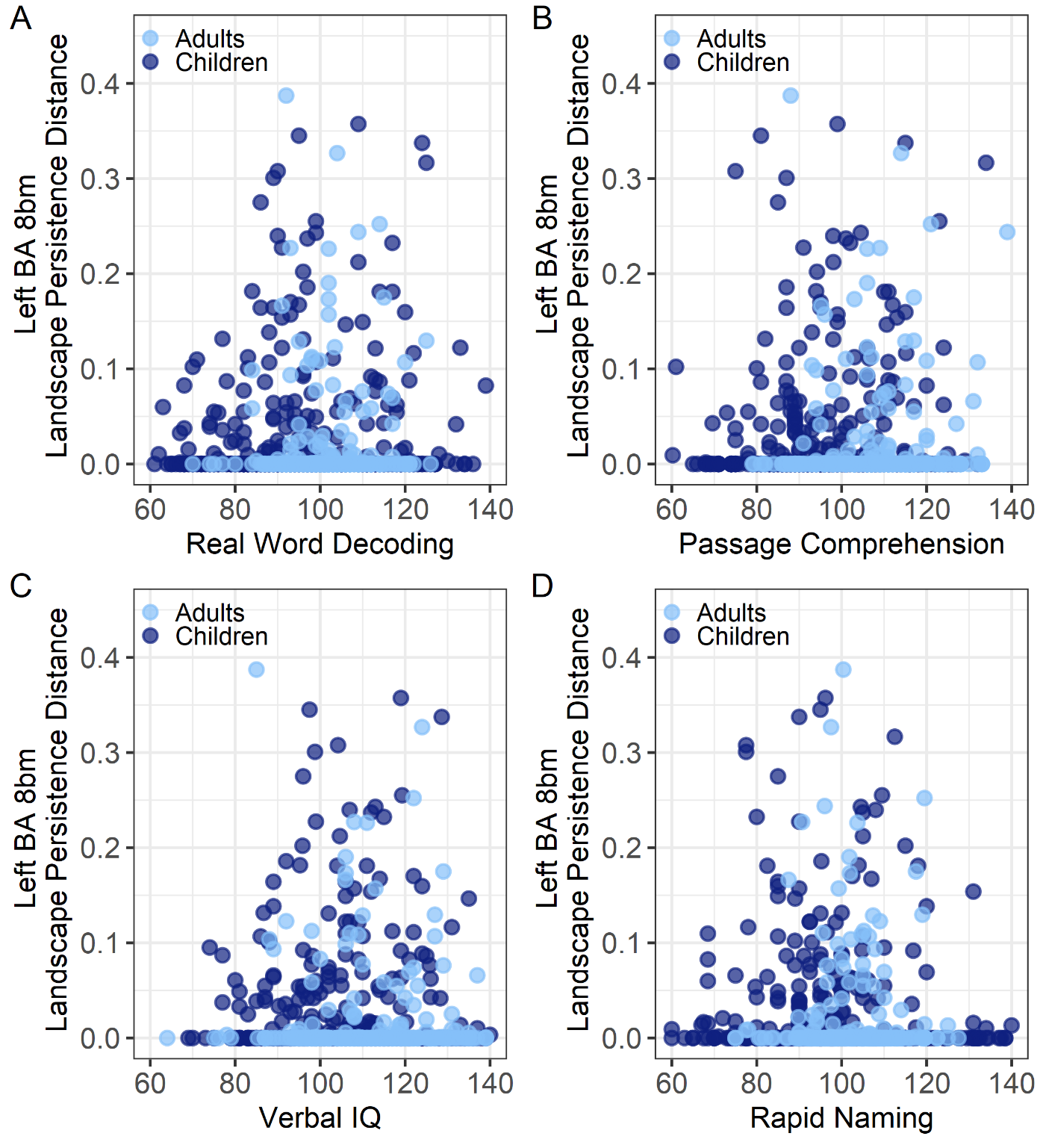
**

**Fig I.** Dorsal cingulate / paracingulate asymmetry associations with real word decoding / reading, passage comprehension, verbal comprehension (Verbal IQ), and rapid automatized naming. These associations exhibited varying effect sizes but where large enough that the asymmetry association with phonological decoding was no longer significant after controlling the variance from the other behavioral measures in phonological decoding. Underlying data and code: https://osf.io/75g9d.


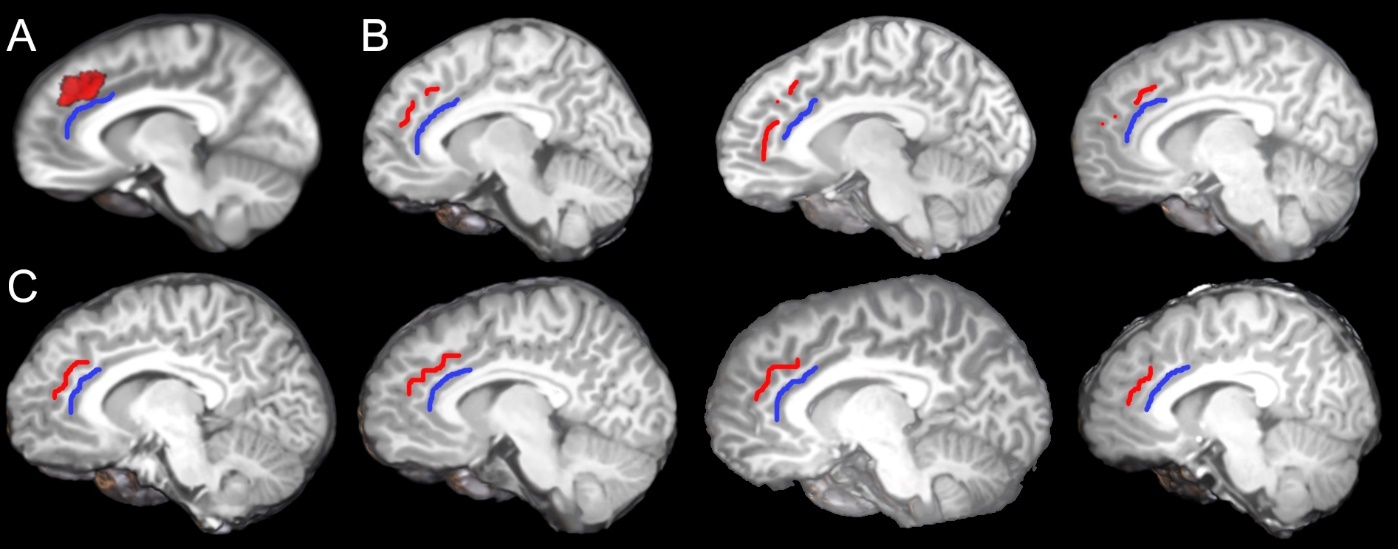


**Fig J.** Participants with the most leftward asymmetries in the Brodmann area (BA) 8bm ROI had a cingulate sulcus (blue line) and a paracingulate sulcus (red line). **A**. Template image with the BA 8bm ROI overlaid (red). **B**. Row of adult images with BA 8bm leftward asymmetries at the 99^th^ percentile. **C**. Row of children with BA 8bm leftward asymmetries at the 99^th^ percentile. Note the parallel labeled sulci, which are largely continuous in the children.

**Table A.** T1-weighted image parameters from the study sites.

| Site | Sample Size | Manufacturer | | Field  Strength  (T) | Image  Dimension  (mm) | Slice  Thickness  (mm) | TR  (msec) | TE  (msec) | Flip  Angle  (deg) |
| --- | --- | --- | --- | --- | --- | --- | --- | --- | --- |
| Pediatric | | |  |  |  |  |  |  |  |
| 1 | 114 | Siemens | | 1.5 | 256 × 256 × 160 | 1.60 | 25.00 | 4.60 | 30 |
| 2 | 44 | Siemens | | 3.0 | 256 × 256 × 124 | 1.20 | 8.7 | 1.90 | 15 |
| 3 | 22 | Siemens | | 3.0 | 176 × 240 × 256 | 0.90 | 2250.00 | 3.96 | 9 |
| 4 | 14 | Siemens | | 3.0 | 128 × 256 × 256 | 1.33 | 6.60 | 2.90 | 8 |
| 5 | 60 | GE | | 1.5 | 124 × 256 × 256 | 1.20 | 11.10 | 2.20 | 25 |
| 6 | 38 | Siemens | | 3.0 | 160 × 256 × 256 | 1.00 | 1600.00 | 3.37 | 15 |
| 7 | 60 | Philips | | 1.5 | 170 × 256 × 256 | 1.00 | 8.02 | 3.69 | 7 |
| 8 | 26 | GE | | 1.5 | 181 × 217 × 181 | 1.00 | 6.00 | 63.00 | -- |
| 9 | 32 | Siemens | | 1.5 | 160 × 256 × 256 | 1.00 | 2000.00 | 3.65 | 8 |
| 10 | 14 | Philips | | 3.0 | 256 × 256 × 120 | 1.10 | 10.36 | 6.00 | 8 |
| Adult | | |  |  |  |  |  |  |  |
| 11 | 1 | GE | | 3.0 | 256 x 256 x 124 | 1.30 | 23.00 | 8.00 | 25 |
|  |  |  | |  |  |  |  |  |  |
| 6/12 | 52 | Siemens | | 3.0 | 160 × 256 × 256 | 1.00 | 1600.00 | 3.37 | 15 |
|  |  |  | |  |  |  |  |  |  |
| 7/13 | 40 | Philips | | 1.5 | 170 x 256 x 256 | 1.00 | 8.02 | 3.69 | 7 |
|  |  |  | |  |  |  |  |  |  |
| 14 | 160 | GE | | 1.5 | 124 x 256 x 256 | 1.20 | 11.00 | 2.20 | 25 |
|  |  |  | |  |  |  |  |  |  |
| 15 | 11 | Philips | | 3.0 | 180 x 256 x 256 | 0.94 | 8.55 | 3.90 | 8 |
|  |  |  | |  |  |  |  |  |  |
| 16 | 1 | Siemens | | 1.5 | 256 x 256 x 120 | 1.10 | 10.36 | 6.00 | 8 |
|  |  |  | |  |  |  |  |  |  |
| 17 | 34 | GE | | 3.0 | 186 x 256 x 256 | 1.00 | 6.02 | 2.19 | 12 |
|  |  |  | |  |  |  |  |  |  |
| 18 | 1 | Philips | | 3.0 | 256 x 256 x 160 | 1.00 | 9.87 | 4.59 | 8 |

De-identified DICOM information was used to collect sequence parameters when available, and from related manuscripts or image header information (flip angle data missing for one site). Relatively long TRs were used for inversion recovery acquisitions (inversion times: site 2 = 900 msec; site 5 = 640 msec; site 12 = 218 msec; site 14 = 180 msec; site 16 = 450 msec). 4a,b- Data were obtained for 2 pediatric studies. 6/12, 7/13- Data were obtained for pediatric and adult studies. The propensity score matching approach for participant selection prioritized matching participants with atypical and typical reading skills within sites, but allowed participants to be matched across research sites, thus resulting in cases from 3 sites where only one participant was included. GE – General Electric; TR – Repetition Time; TE- Echo Time

**Table B**. Significant linear associations between leftward structural asymmetries across the left cerebral hemisphere and phonological decoding (in bold) were not substantively affected after controlling for total brain volume (summed total gray matter and white matter volume).

|  | **Landscape Position** | | | | | | | | | | | | | |
| --- | --- | --- | --- | --- | --- | --- | --- | --- | --- | --- | --- | --- | --- | --- |
|  | | 2 | 3 | 4 | 5 | 6 | 7 | 8 | 9 | 10 | 11 | 12 | 13 | 14 |
| **Children** | | | | | | | | | | | | | | |
| No Total Brain Volume Covariate | | 0.00 | 0.01 | 0.05 | 0.19 | **0.33** | **0.33** | **0.31** | **0.27** | **0.30** | 0.16 | 0.17 | 0.10 | 0.15 |
| Total Brain Volume Covariate | | -0.01 | 0.02 | 0.05 | 0.19 | **0.31** | **0.31** | **0.29** | **0.24** | **0.27** | 0.12 | 0.12 | 0.06 | 0.11 |
| **Adults** | | | | | | | | | | | | | | |
| No Total Brain Volume Covariate | | 0.01 | 0.04 | 0.07 | 0.11 | 0.11 | **0.25** | **0.29** | **0.32** | **0.38** | **0.36** | **0.24** | **0.22** | **0.22** |
| Total Brain Volume Covariate | | 0.01 | 0.04 | 0.17 | **0.25** | **0.24** | **0.38** | **0.42** | **0.46** | **0.49** | **0.46** | **0.31** | **0.28** | **0.28** |

Landscape positions 1 and 15 have no data and thus cannot be analyzed. Controlling for brain volume refers to co-varying or regressing out the effects of total gray and white matter volume from the Word Attack phonological decoding measure before examining its association with the persistent homology data. Bold values were significant (p < 0.05) in both samples with and without the total brain volume covariate.

**Table C**. Linear associations between reading-related measures and left hemisphere persistence distance for the landscape position where the phonological decoding measure was maximally associated with structural asymmetries (Children: Position 7; Adults: Position 10).

|  | | **Children** | | | | **Adults** | | | |
| --- | --- | --- | --- | --- | --- | --- | --- | --- | --- |
|  | *t* | | 95% *CI* | *p* | Cohen's *d* | *t* | 95% *CI* | *p* | Cohen's *d* |
| Phonological Decoding | 3.40 | | 1.29 - 5.52 | 0.0007 | 0.33 | 3.91 | 1.83 - 6.04 | 0.0001 | 0.45 |
| Real Word Reading | 2.97 | | 1.07 - 5.00 | 0.003 | 0.29 | 1.83 | -0.11 - 3.76 | 0.068 | 0.21 |
| Passage Comprehension | 2.43 | | 0.71 - 4.28 | 0.015 | 0.24 | -0.67 | -4.86 – 1.58 | 0.504 | -0.08 |
| Verbal Comprehension | 2.37 | | 0.32 - 4.45 | 0.018 | 0.23 | 0.89 | -1.03 – 2.76 | 0.373 | 0.10 |
| Rapid Naming | -0.38 | | -2.35 - 1.52 | 0.705 | -0.19 | 2.37 | 0.50 - 4.43 | 0.019 | 0.28 |

Statistics were pooled across the 10 imputed datasets.

**Table D**. Descriptive statistics and Pearson correlations for the reading-related variables in the pediatric and adult samples.

| Language Abilities | Sample | Mean (SD) | 1 | 2 | 3 | 4 |
| --- | --- | --- | --- | --- | --- | --- |
| 1: Phonological Decoding | Children | 97.74 (14.61) | - |  |  |  |
| (Word Attack) | Adults | 99.67 (10.10) | - |  |  |  |
|  |  |  |  |  |  |  |
| 2: Real Word Identification | Children | 96.20 (17.37) | 0.86 *** | - |  |  |
| (Word Identification) | Adults | 101.79 (8.69) | 0.60 *** | - |  |  |
|  |  |  |  |  |  |  |
| 3: Reading Comprehension (Passage Comprehension) | Children | 95.37 (15.29) | 0.68 *** | 0.80 *** | - |  |
|  | Adults | 108.38 (12.43) | 0.41 *** | 0.58 *** | - |  |
|  |  |  |  |  |  |  |
| 4: Verbal IQ | Children | 105.85 (14.97) | 0.50 *** | 0.53 *** | 0.62 *** | - |
| (Verbal Comprehension) | Adults | 111.21 (13.05) | 0.40 *** | 0.64 *** | 0.60 *** | - |
|  |  |  |  |  |  |  |
| 5: Rapid Automatized Naming | Children | 97.12 (16.36) | 0.10 * | 0.19 *** | 0.37 *** | 0.20 *** |
|  | Adults | 102.73 (9.34) | 0.41 *** | 0.34 *** | 0.33 *** | 0.08 |

* *p* < 0.05; *** *p* < 0.001; Means (SD) and Correlation coefficients were pooled across the 10 imputed datasets.

**Table E**. Significant inverted-U associations between BA 8bm or BA 6d leftward structural asymmetries and phonological decoding (in bold font) were largely unaffected after controlling for total brain volume (summed total gray matter and white matter volume).

|  | **Landscape Position** | | | | | | | | | | | | | | | | | | | | | | |  |
| --- | --- | --- | --- | --- | --- | --- | --- | --- | --- | --- | --- | --- | --- | --- | --- | --- | --- | --- | --- | --- | --- | --- | --- | --- |
|  | | 2 | 3 | 4 | 5 | 6 | | 7 | | 8 | | 9 | | 10 | | 11 | | 12 | | 13 | | 14 | |  |
| **Children: BA 8bm** | | | | | | | | | | | | | | | | | | | | | | | |  |
| No Total Brain Volume Covariate | | -0.17 | **-0.19** | **-0.24** | **-0.27** | | **-0.3** | | **-0.4** | | **-0.4** | | **-0.24** | | **-0.25** | | **-0.23** | | **-0.23** | | **-0.24** | | **-0.24** | |
| Total Brain Volume Covariate | | -0.10 | -0.15 | **-0.30** | **-0.28** | | **-0.31** | | **-0.32** | | **-0.37** | | **-0.20** | | **-0.19** | | **-0.24** | | **-0.23** | | **-0.25** | | **-0.26** | |
| **Adults: BA 8bm** | | | | | | | | | | | | | | | | | | | | | | | |  |
| No Total Brain Volume Covariate | | **-0.20** | **-0.20** | **-0.20** | **-0.20** | | **-0.20** | | **-0.20** | | **-0.20** | | **-0.20** | | **-0.20** | | **-0.20** | | **-0.20** | | **-0.23** | | **-0.23** | |
| Total Brain Volume Covariate | | **-0.20** | **-0.20** | **-0.20** | **-0.20** | | **-0.20** | | **-0.20** | | **-0.20** | | **-0.20** | | **-0.20** | | **-0.20** | | **-0.20** | | **-0.19** | | **-0.19** | |
| **Children: BA 6d** | |  |  |  |  |  | |  | |  | |  | |  | |  | |  | |  | |  | |  |
| No Total Brain Volume Covariate | | -0.03 | -0.15 | -0.07 | 0.02 | | -0.08 | | **-0.23** | | **-0.20** | | **-0.23** | | -0.19 | | -0.11 | | -0.19 | | -0.13 | | -0.17 | |
| Total Brain Volume Covariate | | -0.14 | -0.17 | -0.06 | -0.11 | | -0.16 | | **-0.22** | | -0.02 | | -0.11 | | -0.17 | | -0.10 | | -0.19 | | -0.1 | | -0.16 | |
| **Adults: BA 6d** | |  |  |  |  | |  | |  | |  | |  | |  | |  | |  | |  | |  | |
| No Total Brain Volume Covariate | | **-0.19** | **-0.2** | **-0.21** | **-0.21** | | **-0.21** | | **-0.21** | | **-0.21** | | **-0.23** | | **-0.23** | | **-0.22** | | **-0.22** | | **-0.22** | | **-0.22** | |
| Total Brain Volume Covariate | | -0.19 | -0.19 | -0.19 | **-0.21** | | **-0.22** | | **-0.22** | | **-0.22** | | **-0.24** | | **-0.26** | | **-0.25** | | **-0.26** | | **-0.26** | | **-0.26** | |
